# Supplementary material for: stClinic dissects clinically relevant niches by integrating spatial multi-slice multi-omics data in dynamic graphs
Source: Nat Commun. 2025 Jun 16;16:5317. doi: 10.1038/s41467-025-60575-x (PMC12170857; doi:10.1038/s41467-025-60575-x)
Supplement: Supplementary file 3 — Reporting Summary [file 41467_2025_60575_MOESM3_ESM.pdf]

nature portfolio

Corresponding author(s): Chunman Zuo and Luonan Chen

Last updated by author(s): May 23, 2025

Reporting Summary

Nature Portfolio wishes to improve the reproducibility of the work that we publish. This form provides structure for consistency and transparency in reporting. For further information on Nature Portfolio policies, see our Editorial Policies and the Editorial Policy Checklist.

Statistics

For all statistical analyses, confirm that the following items are present in the figure legend, table legend, main text, or Methods section.

n/a	Confirmed	
☐	☒	The exact sample size (*n*) for each experimental group/condition, given as a discrete number and unit of measurement
☐	☒	A statement on whether measurements were taken from distinct samples or whether the same sample was measured repeatedly
☐	☒	The statistical test(s) used AND whether they are one- or two-sided<br>*Only common tests should be described solely by name; describe more complex techniques in the Methods section.*
☐	☒	A description of all covariates tested
☐	☒	A description of any assumptions or corrections, such as tests of normality and adjustment for multiple comparisons
☐	☒	A full description of the statistical parameters including central tendency (e.g. means) or other basic estimates (e.g. regression coefficient) AND variation (e.g. standard deviation) or associated estimates of uncertainty (e.g. confidence intervals)
☐	☒	For null hypothesis testing, the test statistic (e.g. *F*, *t*, *r*) with confidence intervals, effect sizes, degrees of freedom and *P* value noted<br>*Give P values as exact values whenever suitable.*
☒	☐	For Bayesian analysis, information on the choice of priors and Markov chain Monte Carlo settings
☒	☐	For hierarchical and complex designs, identification of the appropriate level for tests and full reporting of outcomes
☒	☐	Estimates of effect sizes (e.g. Cohen's *d*, Pearson's *r*), indicating how they were calculated

Our web collection on statistics for biologists contains articles on many of the points above.

Software and code

Policy information about availability of computer code

Data collection	No software was used.
Data analysis	stClinic and all the code for reproducing the analyses and benchmarking are freely available under the MIT License. stClinic is implemented based on python 3.8.5 and R 4.3.2. Other tools and packages used in the data analysis include: anndata 0.9.2, numpy 1.22.3, pandas 2.0.3, scipy 1.10.1, matplotlib 3.7.2, scanpy 1.9.3, umap-learn 0.5.3, louvain 0.8.1, h5py 3.9.0, torch 2.4.0, torchaudio 2.4.0, torchvision 0.19.0, tqdm 4.65.0, hnswlib 0.5.1, rpy2 3.5.1, scikit-learn 1.3.0, scikit-misc 0.2.0, seaborn 0.11.2, lifelines 0.27.8, network 3.1, torch-sparse 0.6.18, torch-scatter 2.1.2, Squidpy 1.2.2, SEDR 1.0.0, STAligner 1.0.0, GraphST 1.1.1, Stitch3D 1.0.3, PRECAST 1.6.4, SLAT 0.3.0, BANKSY 0.99.12, SpaGCN 1.2.7, MultiVI 1.3.0, scVI 1.3.0, peakVI 1.3.0, CellCharter 0.3.4, GLUE 0.3.2, MaxFuse 0.0.2, stClinic 0.0.10, Seurat v4, ggplot2 3.3.6. The codes are publicly available at Zenodo https://zenodo.org/records/15246396 95. The stClinic tool will be maintained and updated at https://github.com/cmzuo11/stClinic.

For manuscripts utilizing custom algorithms or software that are central to the research but not yet described in published literature, software must be made available to editors and reviewers. We strongly encourage code deposition in a community repository (e.g. GitHub). See the Nature Portfolio guidelines for submitting code & software for further information.

Data

Policy information about availability of data

All manuscripts must include a data availability statement. This statement should provide the following information, where applicable:

- Accession codes, unique identifiers, or web links for publicly available datasets
- A description of any restrictions on data availability
- For clinical datasets or third party data, please ensure that the statement adheres to our policy

All datasets used in this manuscript are publicly available. The DLPFC dataset is available from the R package spatialLIBD (http://spatial.libd.org/spatialLIBD/) 42. The 3D hippo dataset can be accessed via the link (https://drive.google.com/drive/folders/10lhz5VY7YfvHrtV40MwaqLmWz56U9eBP?usp=sharing). The VIDC, BAS1, and BAS2 datasets of human Luminal B breast cancer are available on the 10X Genomics Website (https://www.10xgenomics.com/datasets/). The Visium and Xenium datasets of human Her2+ breast cancer are available from the Gene Expression Omnibus (GEO) with GSE243280 [https://www.ncbi.nlm.nih.gov/geo/query/acc.cgi?acc=GSE243280]. The seqFISH and Stereo-seq datasets of mouse embryo are available at (https://marionilab.cruk.cam.ac.uk/SpatialMouseAtlas/) and (https://db.cngb.org/stomics/mosta/download/), respectively. The TNBC dataset containing 43 slices is available from GEO with GSE210616 [https://www.ncbi.nlm.nih.gov/geo/query/acc.cgi?acc=GSE210616], and the corresponding clinical information is available at the website (https://doi.org/10.1158/0008-5472.CAN-22-2682). The CRCLM dataset can be accessed via the link (https://drive.google.com/file/d/1QsQIT0-iwcWBFzUBcLUPKYuSnSBxfaME/view?usp=drive_link). The Brain dataset profiled by spatial ATAC-RNA-seq is accessible from GEO with GSE205055 [https://www.ncbi.nlm.nih.gov/geo/query/acc.cgi?acc=GSE205055]. The E10.5 mouse embryo profiled by Stereo-seq and E11 mouse embryo profiled by Spatial ATAC-seq are available from the link (https://db.cngb.org/stomics/mosta/download/) and GEO with GSE171943 [https://www.ncbi.nlm.nih.gov/geo/query/acc.cgi?acc=GSE171943], respectively. The scRNA-seq datasets of CID4535, TNBC, and CRC samples are available from GEO under accession numbers GSE176078 [https://www.ncbi.nlm.nih.gov/geo/query/acc.cgi?acc=GSE176078], GSE176078 [https://www.ncbi.nlm.nih.gov/geo/query/acc.cgi?acc=GSE176078], and GSE132465 [https://www.ncbi.nlm.nih.gov/geo/query/acc.cgi?acc=GSE132465], respectively. Source data provided for this paper are available at figshare.

Research involving human participants, their data, or biological material

Policy information about studies with human participants or human data. See also policy information about sex, gender (identity/presentation), and sexual orientation and race, ethnicity and racism.

Reporting on sex and gender	Not applicable.
Reporting on race, ethnicity, or other socially relevant groupings	Not applicable.
Population characteristics	Not applicable.
Recruitment	Not applicable.
Ethics oversight	Not applicable.

Note that full information on the approval of the study protocol must also be provided in the manuscript.

Field-specific reporting

Please select the one below that is the best fit for your research. If you are not sure, read the appropriate sections before making your selection.

☒ Life sciences ☐ Behavioural & social sciences ☐ Ecological, evolutionary & environmental sciences

For a reference copy of the document with all sections, see nature.com/documents/nr-reporting-summary-flat.pdf

Life sciences study design

All studies must disclose on these points even when the disclosure is negative.

Sample size	No sample size was calculated. All data utilized in the study were downloaded from the public databases to access the capabilities of stClinic. Specifically, (i) 12 slices of the human DLPFC dataset genrated using Visium technology and 7 consecutive sections of the 3D hippocampal structure genrated using Slide-seq were used to validate the functionality of stClinic in aligning and identifying common tissue structures across various heterogenous datasets; (ii) two slices (BAS1 and BAS2) from the same human Luminal B breast cancer were used to assess the efficiency of stClinic in characterizing intra-tumoral heterogeneity; (iii) 43 slices of triple negative breast cancer, with comprehensive information including age, stage and survival time, were utilized to evaluate stClinic's ability to predict prognosis-related tumor microenvironments (TMEs); (iv) 24 tissue sections from the primary colorectal cancer and liver metastasis dataset were used to assess whether stClnic could identify liver-metastasis-related TMEs; (v) three human Luminal B breast cancer slices (IDC, BAS1, and BAS2) were used to evaluate the label transfer efficiency of stClinic; (vi) mouse brain coronal section profiling ATAC-seq and RNA-seq data were employed to evaluate stClinic's ability to integrate multi-omics data from the same slice; (vii) two mouse slices—E10.5 from Stereo-seq and E11 from Spatial ATAC-seq—were utilized to assess stClinic's capability to fuse multi-omics data from different slices; (viii) E8.75 seqFISH and E9.5 Stereo-seq mouse embryo samples, as well as Visium and Xenium slices from the same breast cancer, were used to evaluate stClinic's ability to integrate slices from different technologies.

Data exclusions	All spots/cells and genes of each dataset was used, and no exclusion was done prior to analysis. We performed quality control and gene selection for spatially resolved transcriptomics data based on established standards in the filed.
Replication	We did not conduct biological or technical replications. All data were obtained from the public domain. The reproducibility details are outlined in the methods or GitHub section. To assess the stability and reproducibility of stClinic, we ran the algorithm five times on the dataset using the same parameters, and calculated the mean of the results.
Randomization	The allocation was random.
Blinding	Blinding is irrelevant as there was no data collection involved in the present study. Data information was included in the original publication.

Reporting for specific materials, systems and methods

We require information from authors about some types of materials, experimental systems and methods used in many studies. Here, indicate whether each material, system or method listed is relevant to your study. If you are not sure if a list item applies to your research, read the appropriate section before selecting a response.

Materials & experimental systems

n/a	Involved in the study
☒	☐ Antibodies
☒	☐ Eukaryotic cell lines
☒	☐ Palaeontology and archaeology
☒	☐ Animals and other organisms
☒	☐ Clinical data
☒	☐ Dual use research of concern
☒	☐ Plants

Methods

n/a	Involved in the study
☒	☐ ChIP-seq
☒	☐ Flow cytometry
☒	☐ MRI-based neuroimaging

Plants

Seed stocks	Not applicable.
Novel plant genotypes	Not applicable.
Authentication	Not applicable.
