# Supplementary material for: stClinic dissects clinically relevant niches by integrating spatial multi-slice multi-omics data in dynamic graphs
Source: Nat Commun. 2025 Jun 16;16:5317. doi: 10.1038/s41467-025-60575-x (PMC12170857; doi:10.1038/s41467-025-60575-x)
Supplement: Supplementary file 1 — Supplementary Information [file 41467_2025_60575_MOESM1_ESM.pdf]

Supplementary information of
stClinic dissects clinically relevant niches by integrating spatial multi-slice multi-omics data in dynamic graphs

Chunman Zuo[1,2,3,4*], Junjie Xia[2], Yupeng Xu[2], Ying Xu[5], Pingting Gao[3], Jing Zhang[6], Yan Wang[7], Luonan Chen[8,9,10,11,12*]

[1] School of Life Sciences, Sun Yat-sen University, Guangzhou 510275, China

[2] Institute of Artificial Intelligence, Donghua University, Shanghai 201620, China

[3] Shanghai Collaborative Innovation Center of Endoscopy, Fudan University, Shanghai 200032 China

[4] Key Laboratory of Symbolic Computation and Knowledge Engineering of Ministry of Education, Jilin University, Changchun 130022, China

[5] System Biology Lab for Metabolic Reprogramming, Department of Human Genetics and Cell Biology, School of Medicine, Southern University of Science and Technology, Shenzhen 518055, China

[6] Department of Pathology, Changzheng Hospital, Secondary Military Medical University, Shanghai 200003, China

[7] College of Computer Science and Technology, Jilin University, Changchun 130022, China

[8] School of Mathematical Sciences, Shanghai Jiao Tong University, Shanghai 200240, China

[9] School of AI, Shanghai Jiao Tong University, Shanghai 200240, China

[10] Key Laboratory of Systems Biology, Shanghai Institute of Biochemistry and Cell Biology, Center for Excellence in Molecular Cell Science, Chinese Academy of Sciences, Shanghai 200031, China

[11] Key Laboratory of Systems Health Science of Zhejiang Province, School of Life Science, Hangzhou Institute for Advanced Study, University of Chinese Academy of Sciences, Chinese Academy of Sciences, Hangzhou 310024, China

[12] West China Biomedical Big Data Center, Med-X center for informatics, West China Hospital, Sichuan University, Chengdu 610041, China

* To whom correspondence should be addressed.

Email: zuochm@mail.sysu.edu.cn; lnchen@sjtu.edu.cn

Supplementary Notes

Supplementary Note1. Evaluation of histological and transcriptomic similarities of the same cluster across slices

To comprehensively evaluate whether clusters from different slices are identical, we introduced novel computational methods to assess both histological and transcriptomic similarities between spots within the same clusters. Specifically, (1) we employed CONCH [1], a foundation model pretrained on extensive histology data, to extract histological features from spots. These features were projected into a shared space across different slices, and cosine similarity was calculated to assess the histological similarity of spots within the same clusters; (2) given the challenges of directly measuring transcriptomic similarity due to batch effects, we focused on known layer-specific genes that should ideally be over-expressed within specific clusters rather than across multiple clusters (Supplementary Table 3). We used the Gini index (GI) to evaluate the expression inequality of these layer-specific genes across clusters, providing a measure of transcriptional similarity between spots across slices.

Our analysis showed that both stClinic and SEDR perform comparably in terms of histological similarity within clusters, outperforming other methods. However, stClinic achieves the highest GI score, indicating superior transcriptional alignment. These findings (Supplementary Fig.5b), combined with the statistical assessment in Fig.2e, demonstrated that stClinic effectively aligns identical clusters across different slices.

Supplementary Note2. Evaluation of cell compositions consistency within the same cluster across different patients

To minimize the influence of background heterogeneity between patients, we proposed a ranking-based approach to enhance the comparison of cell compositions. Specifically, we (i) estimated the proportions of different cell types at each spot using GraphST [2]; (ii) considered the ranking of these proportions within each cluster for every patient as the cell composition values, and (iii) refined these values by calculating their differences from the cell composition values of the same cluster between the two samples. These

refined values were then used in hierarchical cluster analysis, providing a more consistent and accurate comparison.

Supplementary Note3. Evaluation of prediction robustness in stClinic using different seeds

To evaluate the robustness of our predictions, we assessed the stability of the clustering results by running VGAE with different random seeds and comparing the outcomes with those from another graph-based model, STAligner. As anticipated, increasing the number of clusters introduced some variability in cluster assignments across different seeds, indicating that the model's results can be influenced by seed selection.

To specifically evaluate the consistency of our key findings—cluster 10 (highest positive weight) and cluster 6 (lowest negative weight)—we repeated the VGAE and UMAP processes five times with different random seeds. On average, 75% of the spots in cluster 10 and 92% of the spots in cluster 6 were consistently identified as positive and negative spots, respectively. These results demonstrated that, while some variability is expected due to the nature of deep learning models, the core predictions of stClinic remain robust and reproducible across different random seeds (Supplementary Fig.33).

Supplementary Figures

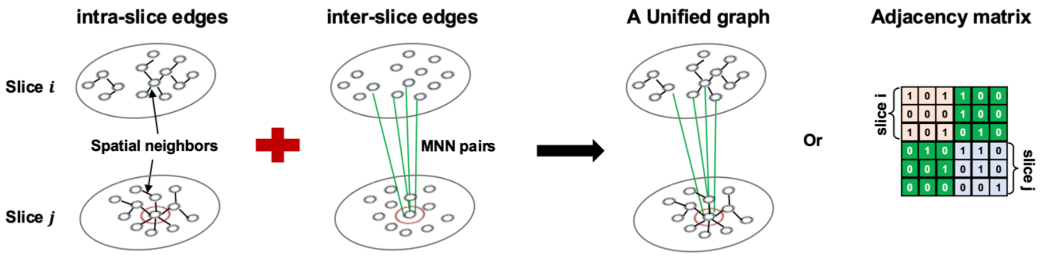

Supplementary Figure 1. Construction of the adjacency matrix (unified graph). For intra-slice edges, stClinic defines neighbors based on a specified radius or the k-nearest neighbor method. For inter-slice edges, neighboring cells/spots are identified as mutual nearest neighbors (MNN) based on feature similarity. The adjacency matrix is formed by combining these two types of edges.

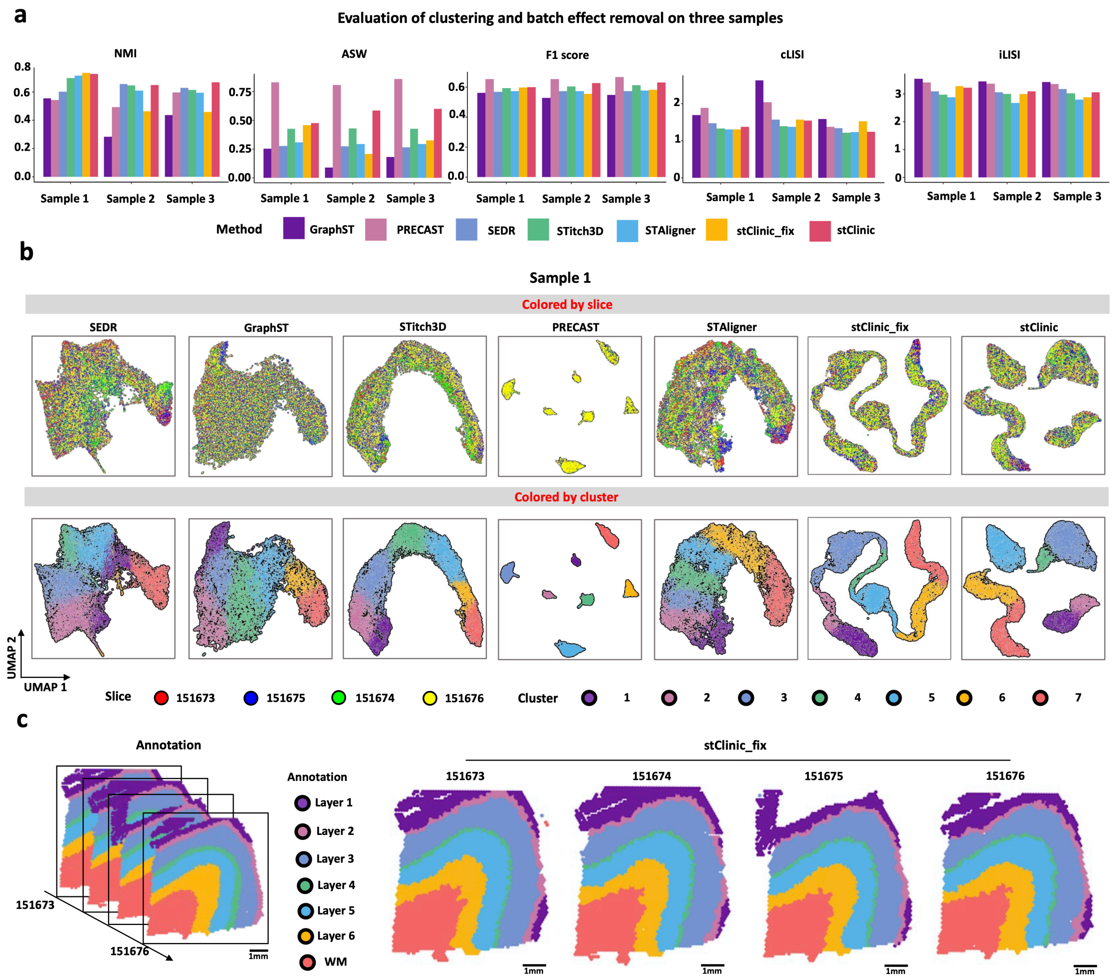

Supplementary Figure 2. Method comparison on the human DLPFC dataset. a Comparison of the seven methods (SEDR, GraphST, STitch3D, PRECAST, STAligner, stClinic_fix, and stClinic) regarding clustering accuracy (NMI and ASW) and batch-effect correction (F1 score, cLISI and iLISI), on three samples. **b** UMAP visualization of latent features by SEDR, GraphST, STitch3D, PRECAST, STAligner, stClinic_fix, and stClinic across all four slices: 151673-151676 of Sample 1. In the top and bottom panels, the colors represent different slices and clusters, respectively. **c** Spatial domains detected by stClinic_fix on the Sample 1, with manual annotation as a reference. Source data are provided as a Source Data file.

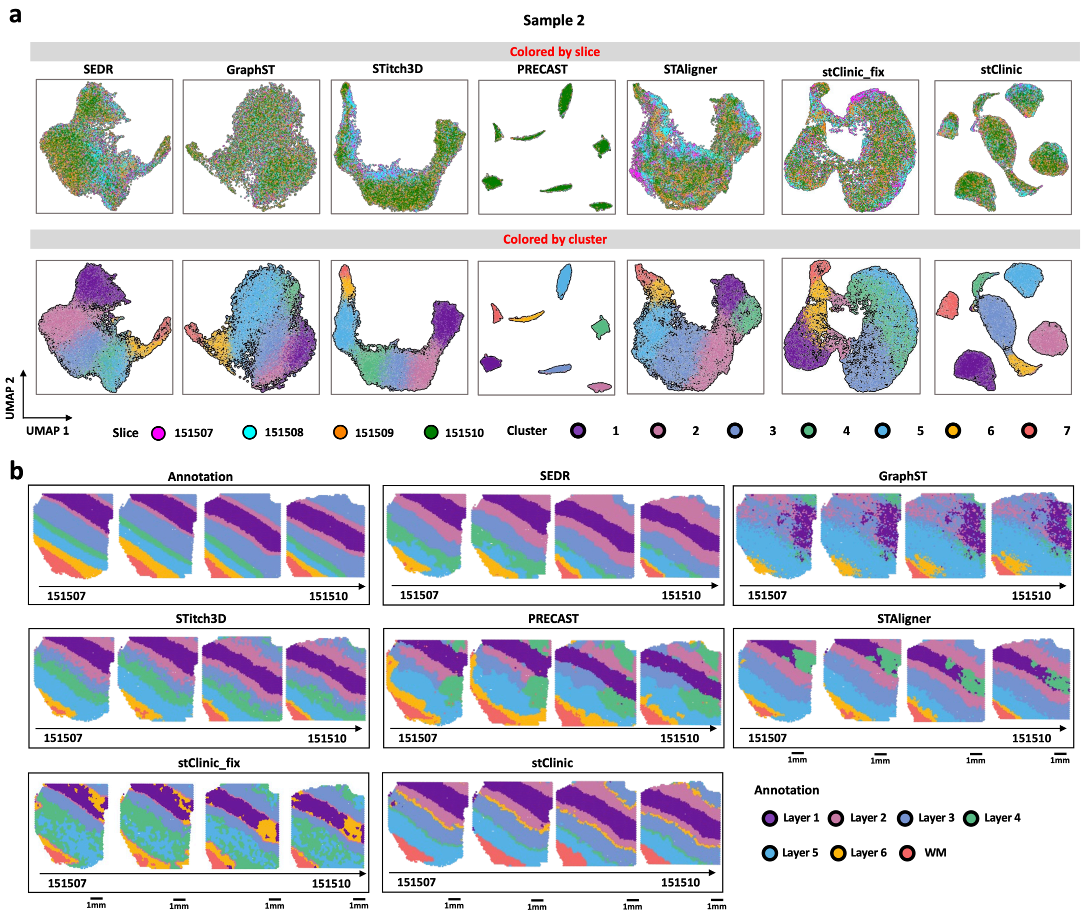

Supplementary Figure 3. Method comparison on Sample 2 of the human DLPFC dataset. a UMAP visualization of latent features by SEDR, GraphST, STitch3D, PRECAST, STAligner, stClinic_fix, and stClinic across all four slices: 151507-151510 of Sample 2. In the top and bottom panels, the colors represent different slices and clusters, respectively. **b** Spatial domains identified by the seven methods (SEDR, GraphST, STitch3D, PRECAST, STAligner, stClinic_fix, and stClinic) on Sample 2, with manual annotation as a reference. Source data are provided as a Source Data file.

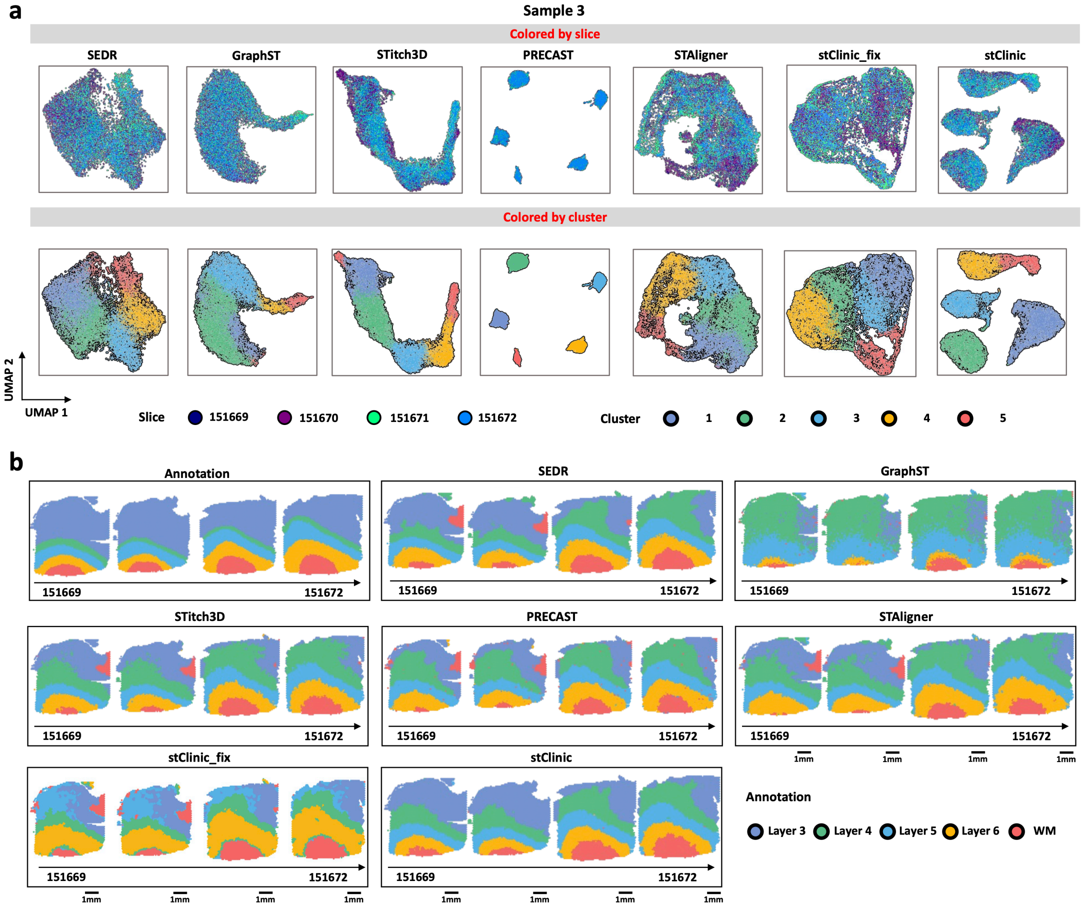

Supplementary Figure 4. Method comparison on Sample 3 of the human DLPFC dataset. a UMAP visualization of the latent features by SEDR, GraphST, STitch3D, PRECAST, STAligner, stClinic_fix, and stClinic across all four slices: 151669-151672 of the Sample 3. In the top and bottom panels, the colors represent different slice and cluster, respectively. **b** Spatial domains identified by the seven methods (SEDR, GraphST, STitch3D, PRECAST, STAligner, stClinic_fix, and stClinic) on the Sample 3, with manual annotation as a reference. Source data are provided as a Source Data file.

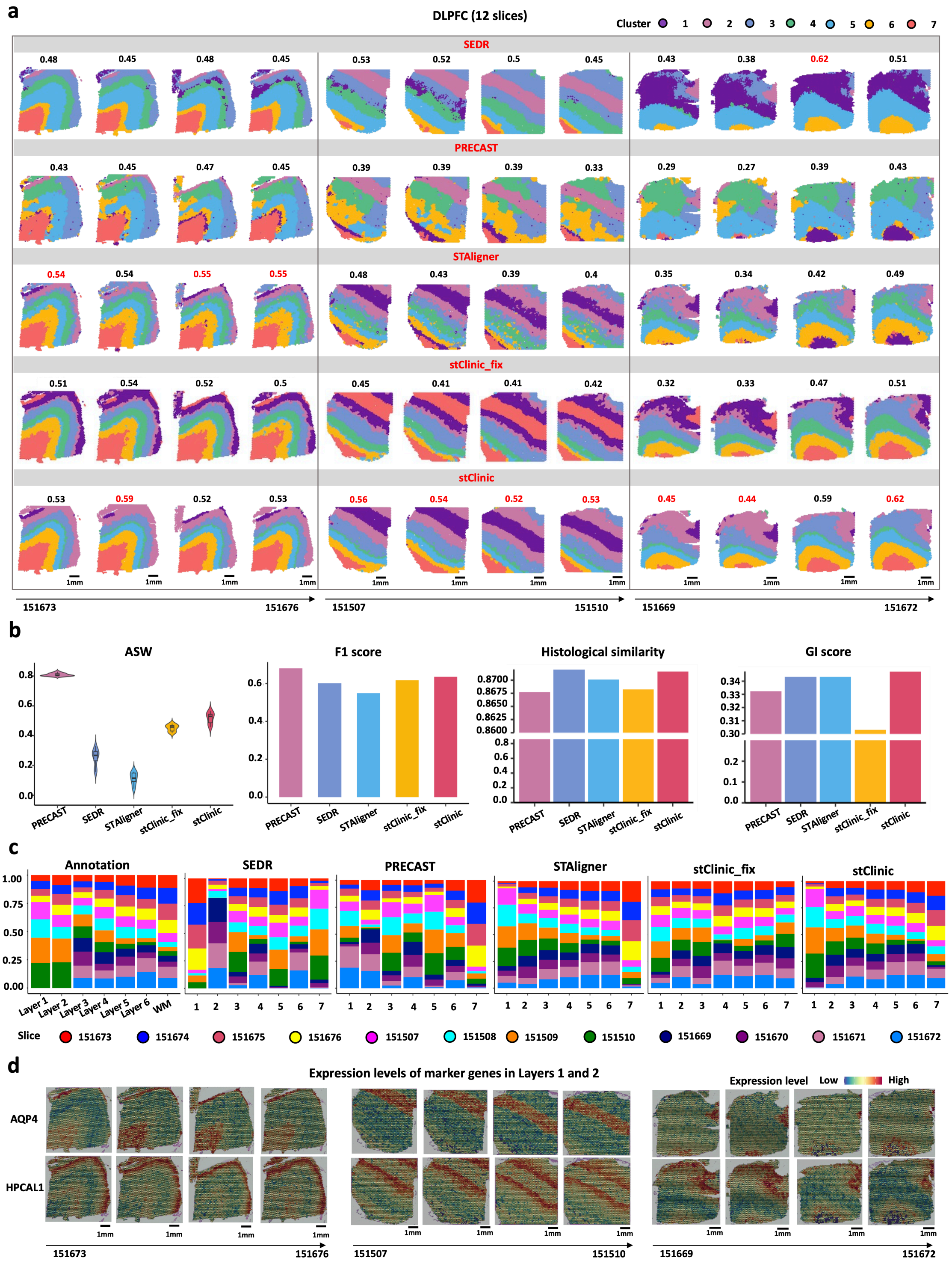

Supplementary Figure 5. Method comparison on $N = 12$ slices of the human DLPFC dataset. a The Spatial domains identified by SEDR, PRECAST, STAligner, stClinic_fix, and stClinic. ARI scores for each method on each slice are displayed above the figure, with the highest values highlighted in red. **b** Comparison of the five methods (SEDR, PRECAST, STAligner, stClinic_fix, and stClinic) in terms of clustering

accuracy (ASW), simultaneous cluster separation and slice mixing (F1 score), histological similarity, and marker gene specificity (GI score), illustrated by violin plots and three bar plots, respectively. **c** Bar chart showing slice compositions within each cluster predicted by each method, with annotations provided for comparison. **d** Spatial expression levels of marker genes in Layer 1 (*AQP4*) and Layer 2 (*HPCAL1*) on 12 slices. Source data are provided as a Source Data file.

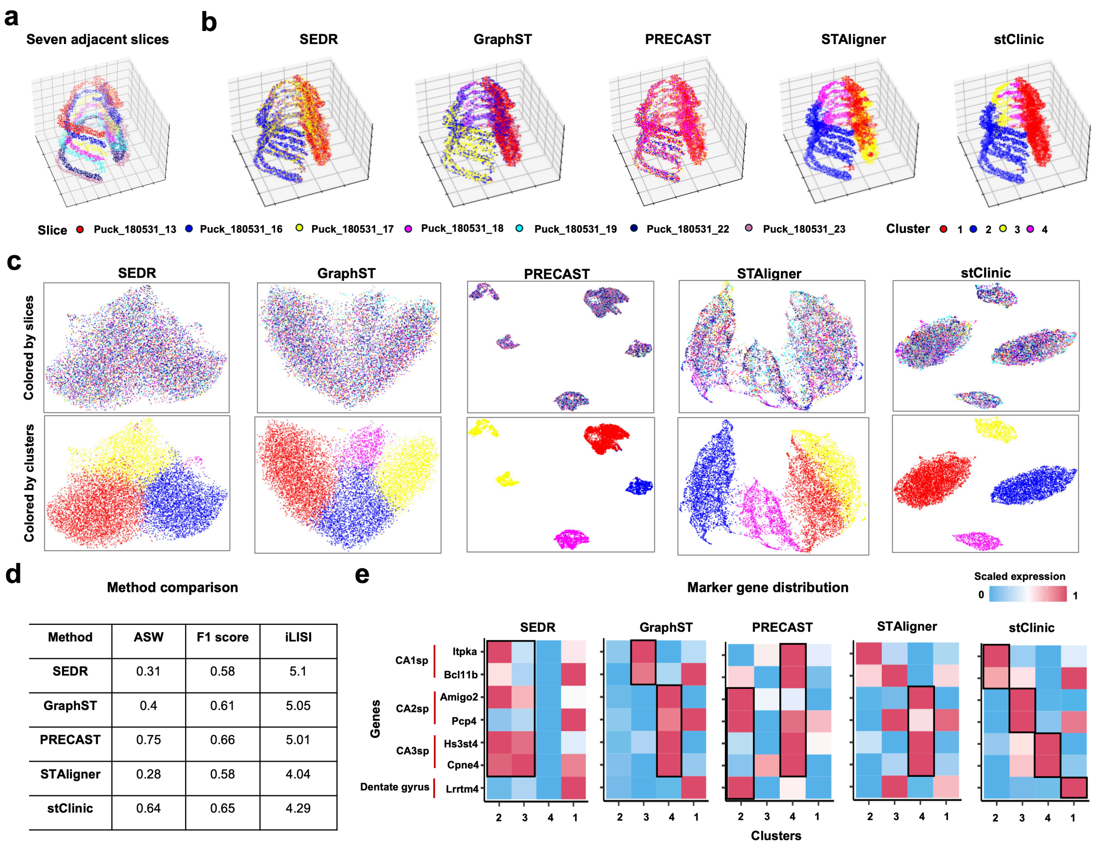

Supplementary Figure 6. Method comparison on seven consecutive sections of the 3D hippocampal structure profiled by Slide-seq. a Visualization of 3D hippocampal structure, stacked from seven aligned consecutive slices. Each color represents one slice. **b** Spatial domains identified by SEDR, GraphST, PRECAST, STAligner, and stClinic, respectively. Each color represents a cluster. **c** UMAP visualization of features by SEDR, GraphST, PRECAST, STAligner, and stClinic, respectively. For each method, the left panel shows color-coded slices, while right panel displays color-coded clusters. **d** Comparison of the five methods (SEDR, GraphST, PRECAST, STAligner, and stClinic) based on cluster separation (ASW), slice mixing (iLISI), and simultaneous cluster separation and slice mixing (F1 score). **e** Mean expression levels of marker genes across spatial clusters identified by SEDR, GraphST, PRECAST, STAligner, and stClinic, with mean expressions scaled by columns. Source data are provided as a Source Data file.

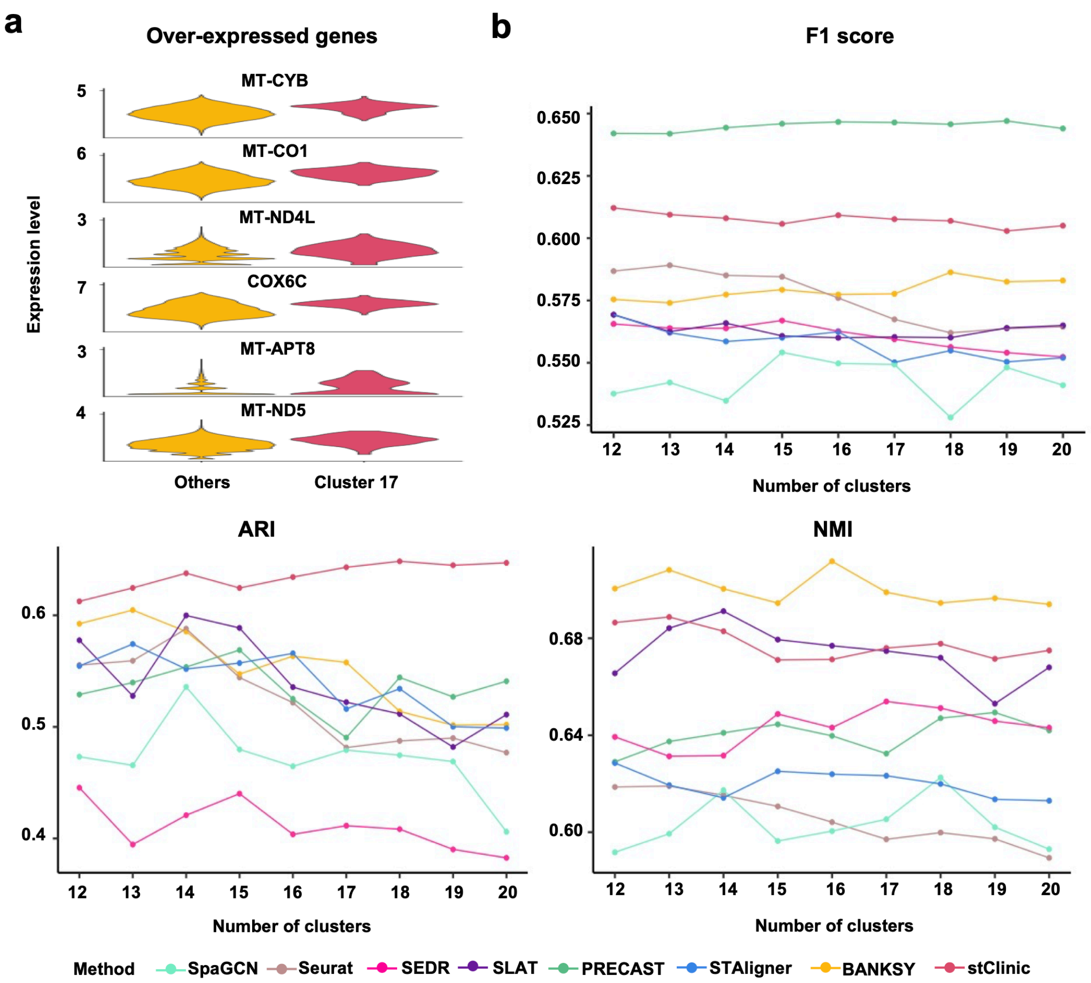

Supplementary Figure 7. Comparison of methods for the integrative analysis of BAS1 and BAS2 slices. a Over-expressed genes in cluster 17 compared to other clusters. **b** Performance evaluation of batch-effect correction (F1 score) and clustering accuracy (ARI and NMI) across varying numbers of clusters (12 to 20). Source data are provided as a Source Data file.

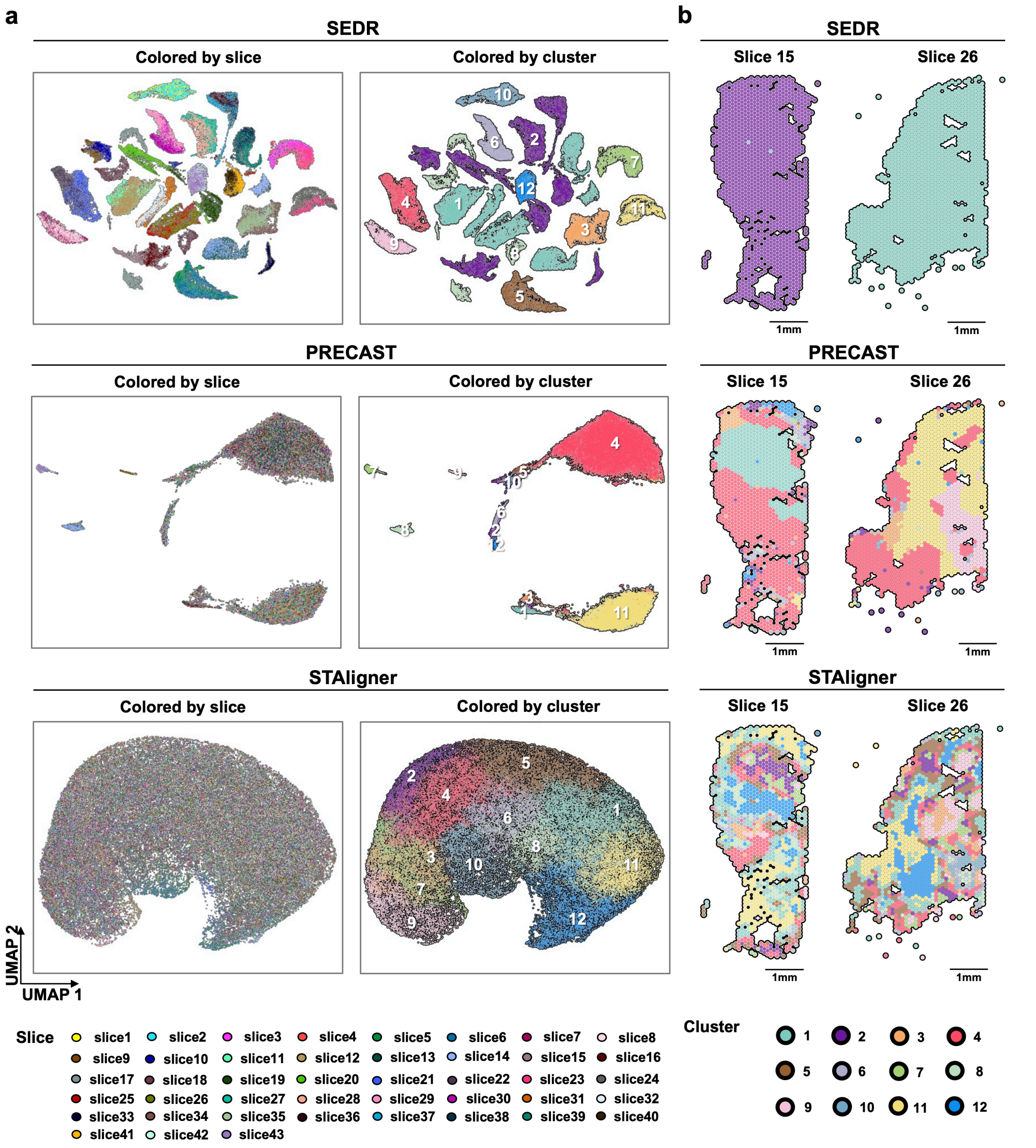

Supplementary Figure 8. Method comparison on 43 slices of the TNBC dataset. a UMAP visualization of the latent features by SEDR, PRECAST, and STAligner on 43 slices, where the colors in the left and right panels indicate slices and clusters, respectively. **b** Spatial domains identified by SEDR, PRECAST, and STAligner on slices 15 and 26. Source data are provided as a Source Data file.

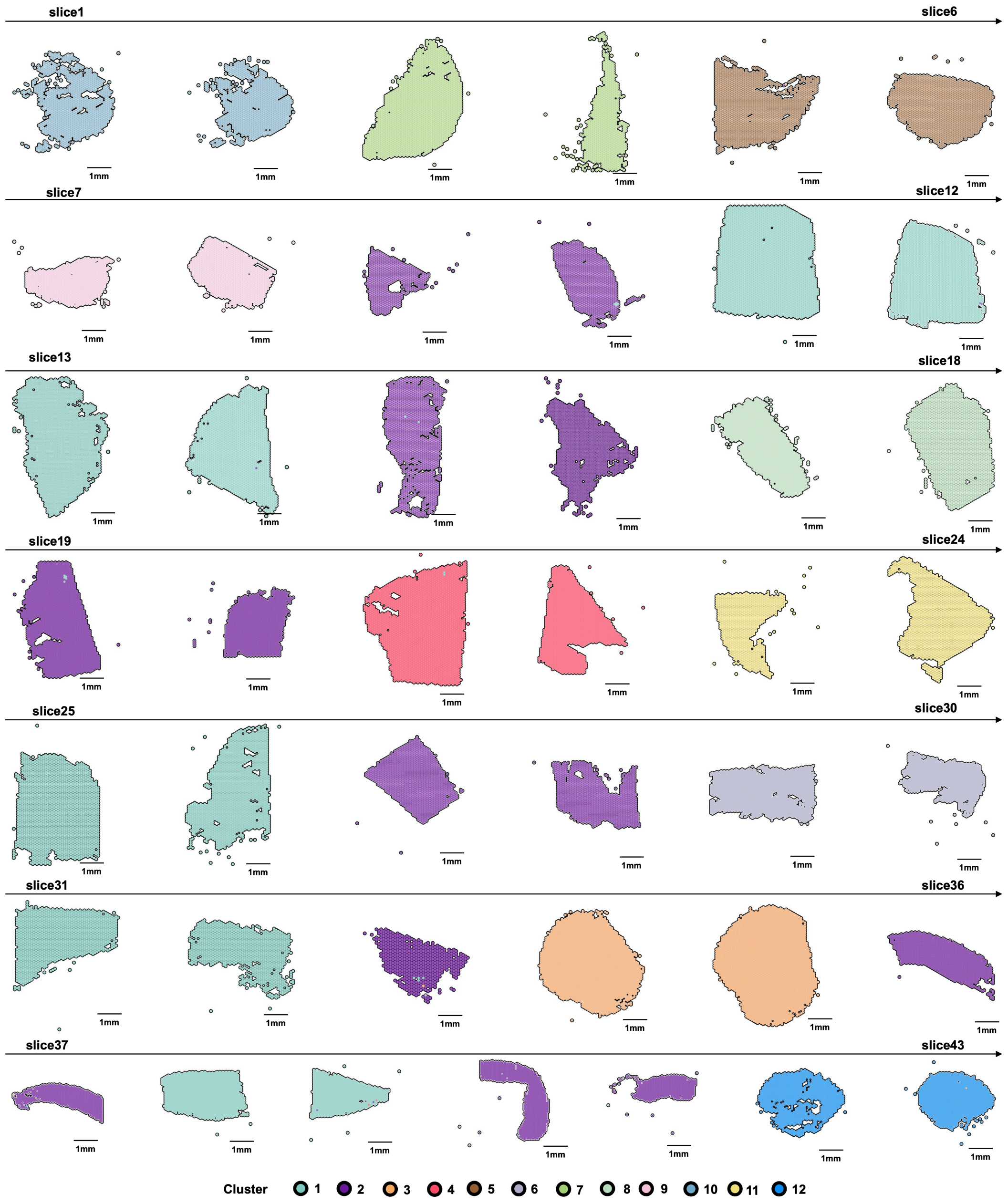

Supplementary Figure 9. Spatial domains identified by SEDR on 43 TNBC slices.

Source data are provided as a Source Data file.

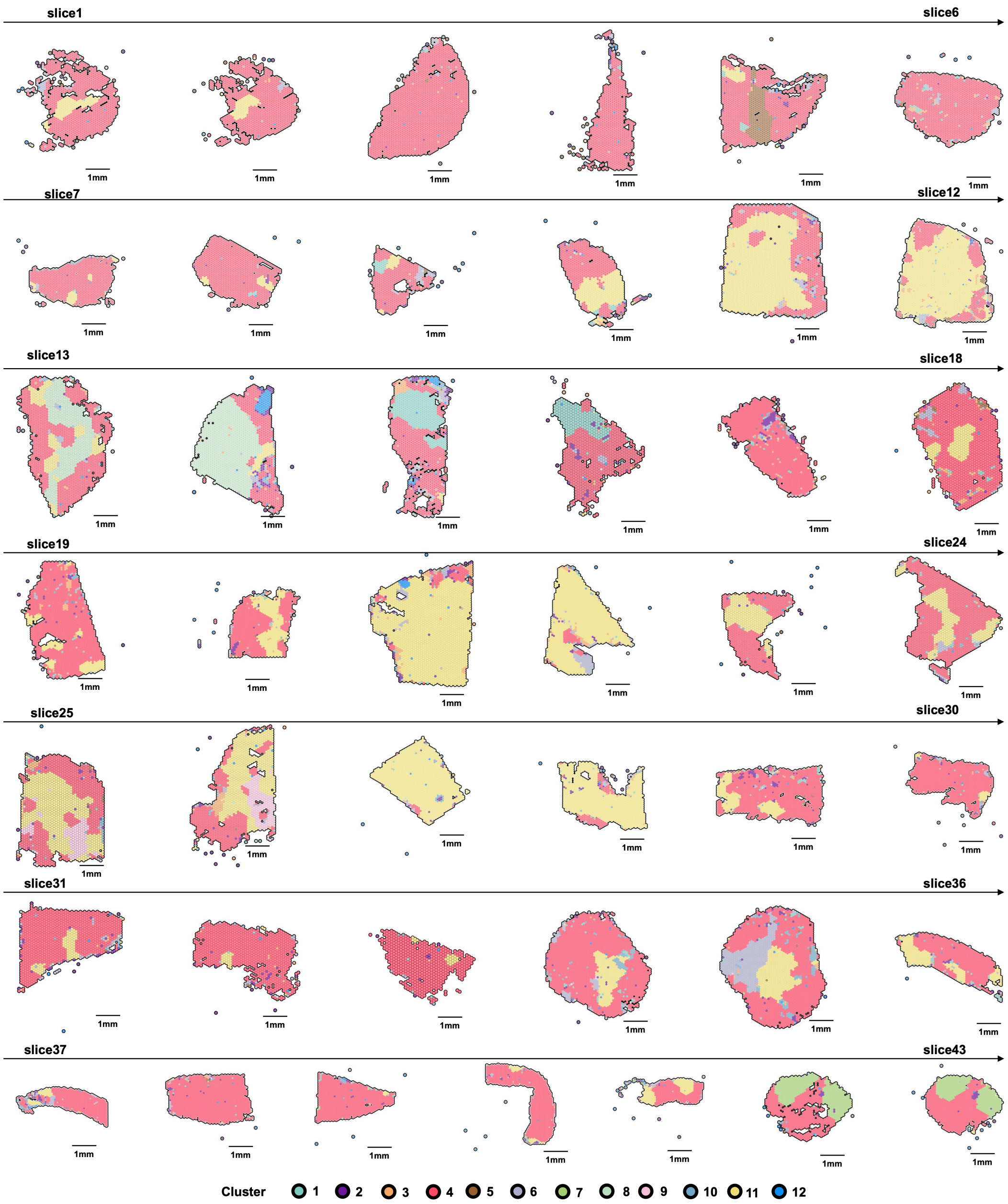

Supplementary Figure 10. Spatial domains identified by PRECAST on 43 TNBC slices. Source data are provided as a Source Data file.

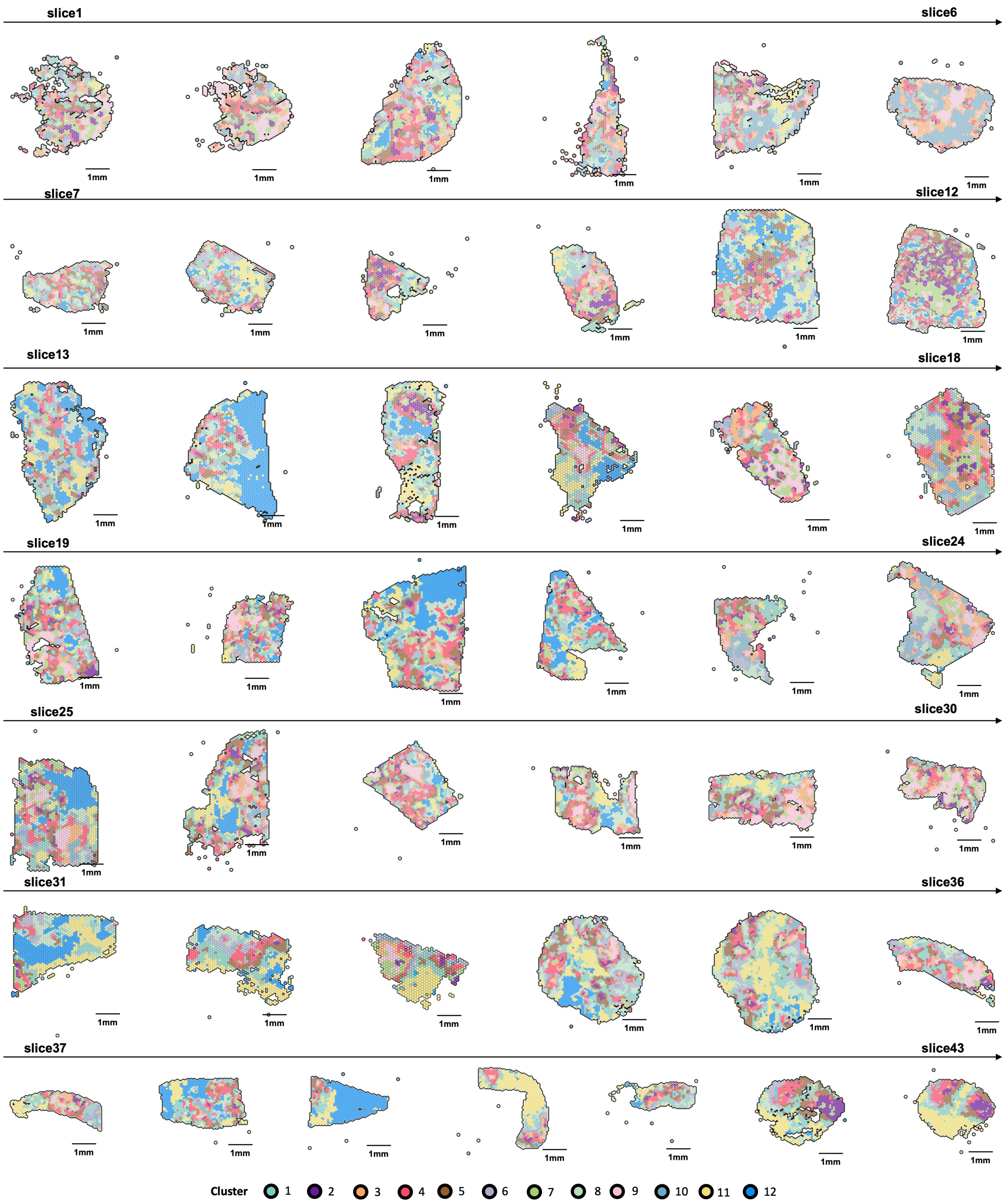

Supplementary Figure 11. Spatial domains identified by STAligner on 43 TNBC slices. Source data are provided as a Source Data file.

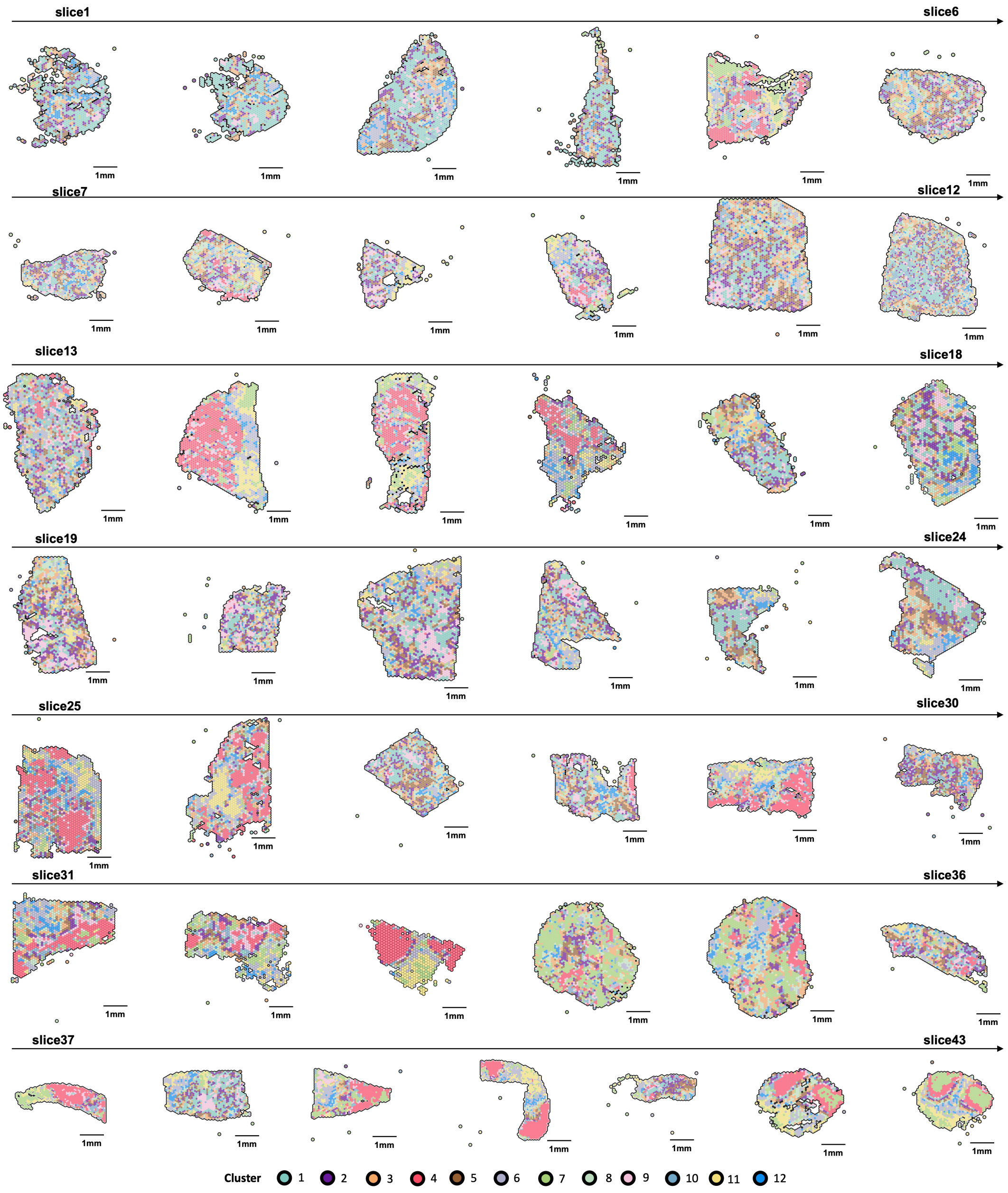

Supplementary Figure 12. Spatial domains identified by stClinic on 43 TNBC slices. Source data are provided as a Source Data file.

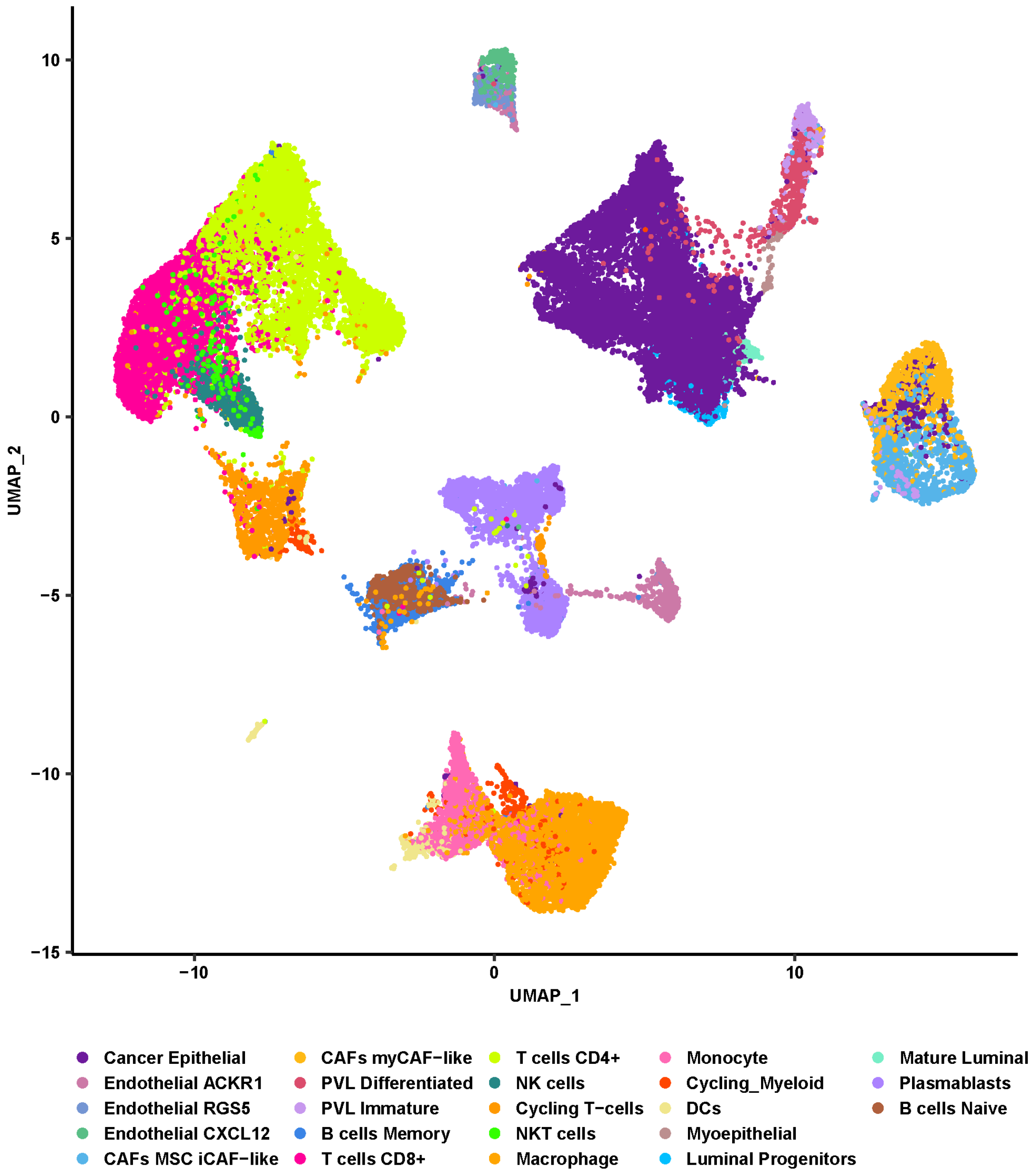

Supplementary Figure 13. UMAP visualization of scRNA-seq data of 42,512 cells from 10 TNBC samples. Each color indicates one cell type. Source data are provided as a Source Data file.

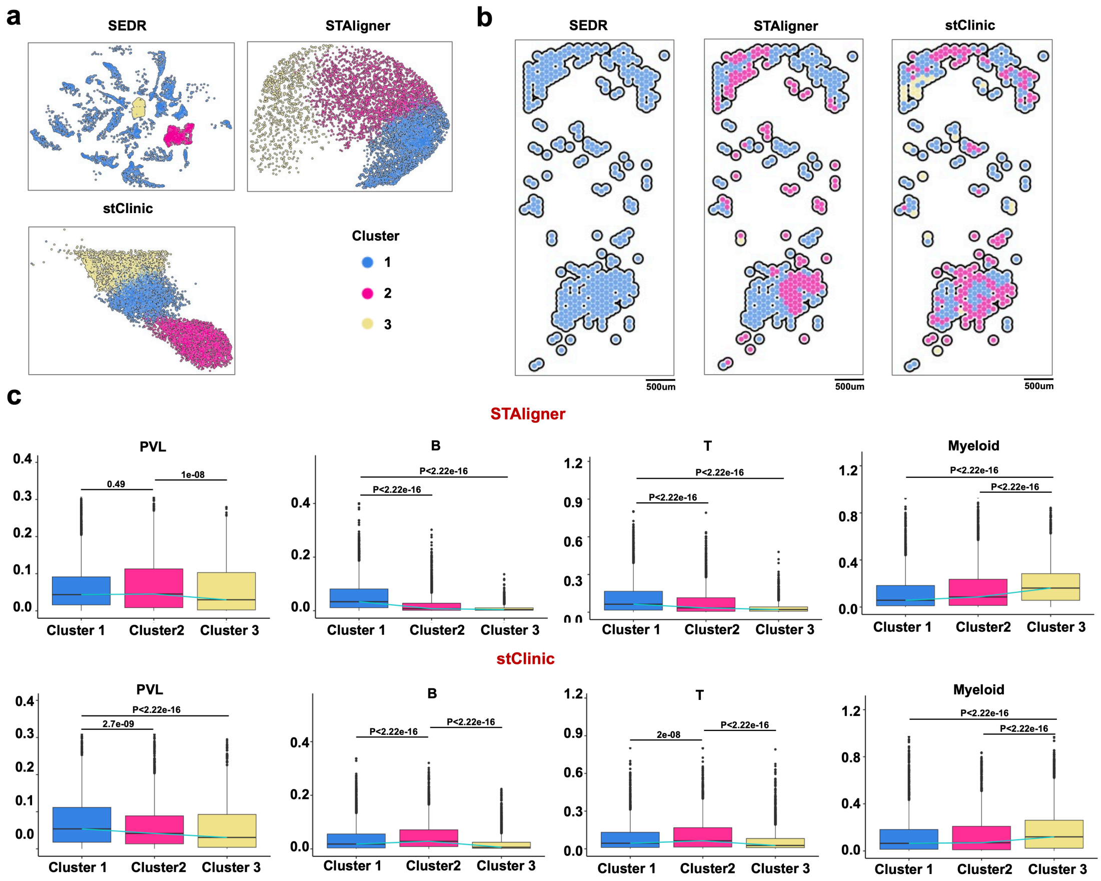

Supplementary Figure 14. Sub-clustering analysis of spots from the dense lymphoid and fibrosis regions in the human TNBC dataset. a Sub-clustering analysis of spot embeddings generated by three graph-based models: SEDR, STAligner, and stClinic. **b** Spatial distribution of three sub-clusters identified by three methods on slice 15. **c** Boxplots showing cell type proportions across the three sub-clusters by STAligner (top) and stClinic (bottom). Each color indicates one sub-cluster. The p-value was computed using the Wilcox rank-sum test. 2.22e-16 represents a very small numbers, effectively close to zero. For STAligner, the number of cells in cluster 1, cluster 2, and cluster 3 is 2,749, 2,678, and 2,142, respectively. For stClinic, the number of cells in cluster 1, cluster 2, and cluster 3 is 3,976, 2,655, and 938, respectively. For each boxplot, the center line, box limits and whiskers separately indicate the median, upper and lower quartiles and 1.5 × interquartile range. Source data are provided as a Source Data file.

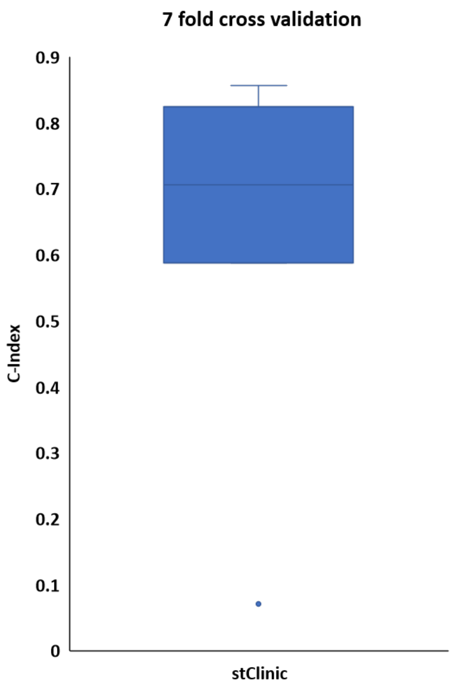

Supplementary Figure 15. Boxplot showing the C-Index predicted by $N = 7$ test sets. The center line, box limits and whiskers for boxplot separately indicate the median, upper and lower quartiles and 1.5 × interquartile range. Source data are provided as a Source Data file.

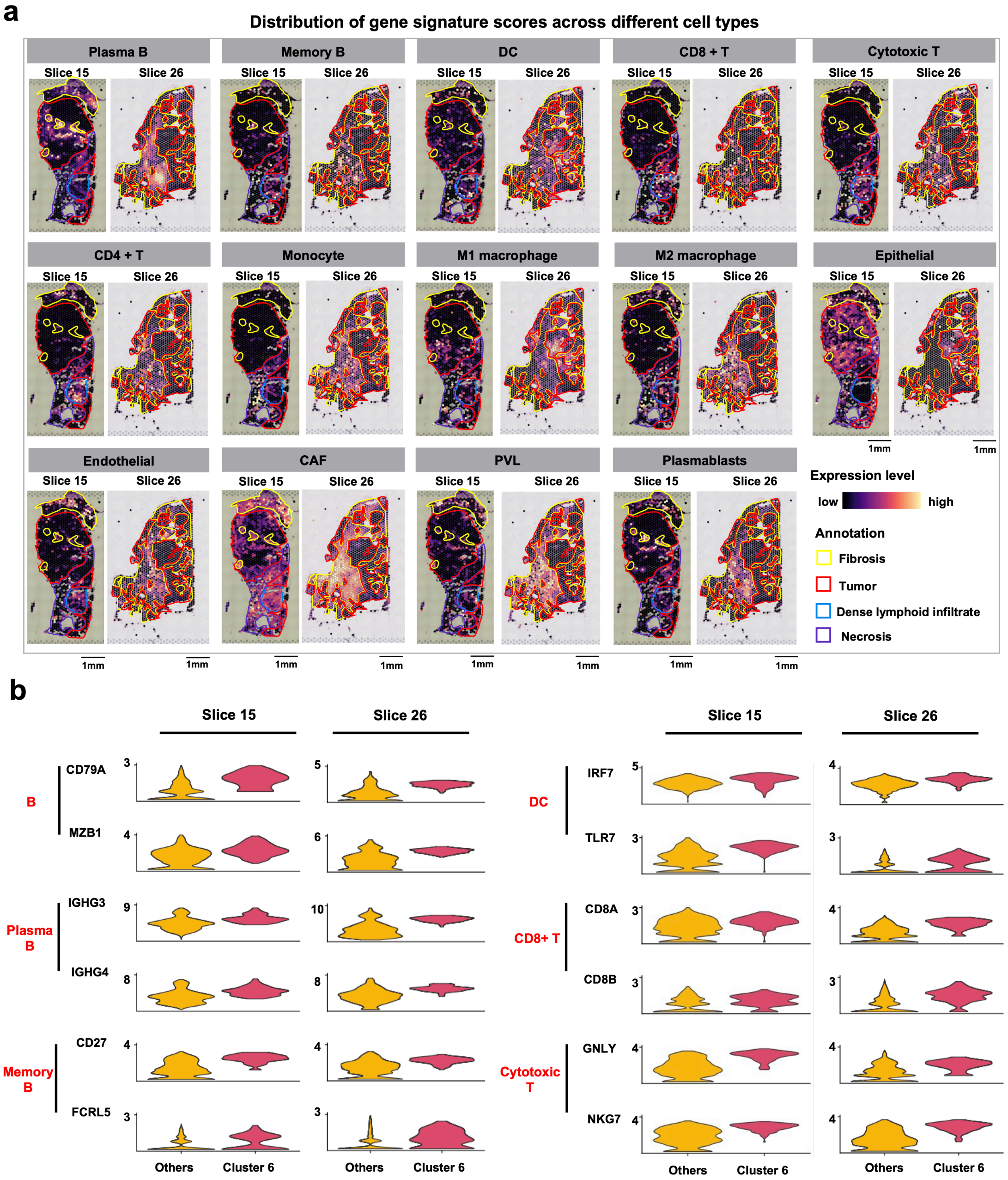

Supplementary Figure 16. Distribution of marker genes for different cell types in the SRT data. a Spatial feature plots of gene signature score for Plasma B cells (*IGHG1*, *IGHG2*, *IGHG3*, and *IGHG4*), Memory B cells (*CD27*, and *FCRL5*), CD8 + T cells (*CD8A*, and *CD8B*), Cytotoxic T cells (*GNLY*, and *NKG7*), CD4 + T cells (*IL7R*, *CCR7*, and *CD4*), DC (*IRF7*, and *LAMP3*), Monocyte (*FCGR3A*), M1 macrophage (*CXCL10*), M2 macrophage (*EGR1*, and *SIGLEC1*), Epithelial (*CDH1*, *KRT19*, *EPCAM*, and *CLDN7*), Endothelial (*CDH5*, *PECAM1*, *VWF*, and *ENG*), CAF (*COL1A1*, *COL1A2*, *COL3A1*, *DCN*, and *MMP2*), PVL (*ACTA2*, and *PDGFRB*), Plasmablasts (*JCHAIN*) on

slices 15 and 26 of the TNBC dataset. **b** Violin plot showing the expression levels of marker genes for B cells (*CD79A*, and *MZB1*), Plasma B cells (*IGHG3*, and *IGHG4*), Memory B cells (*CD27*, and *FCRL5*), DC (*IRF7*, and *TLR7*), CD8+ T cells (*CD8A*, and *CD8B*), and Cytotoxic T cells (*GNLY*, and *NKG7*) across cluster 6 and other groups. Source data are provided as a Source Data file.

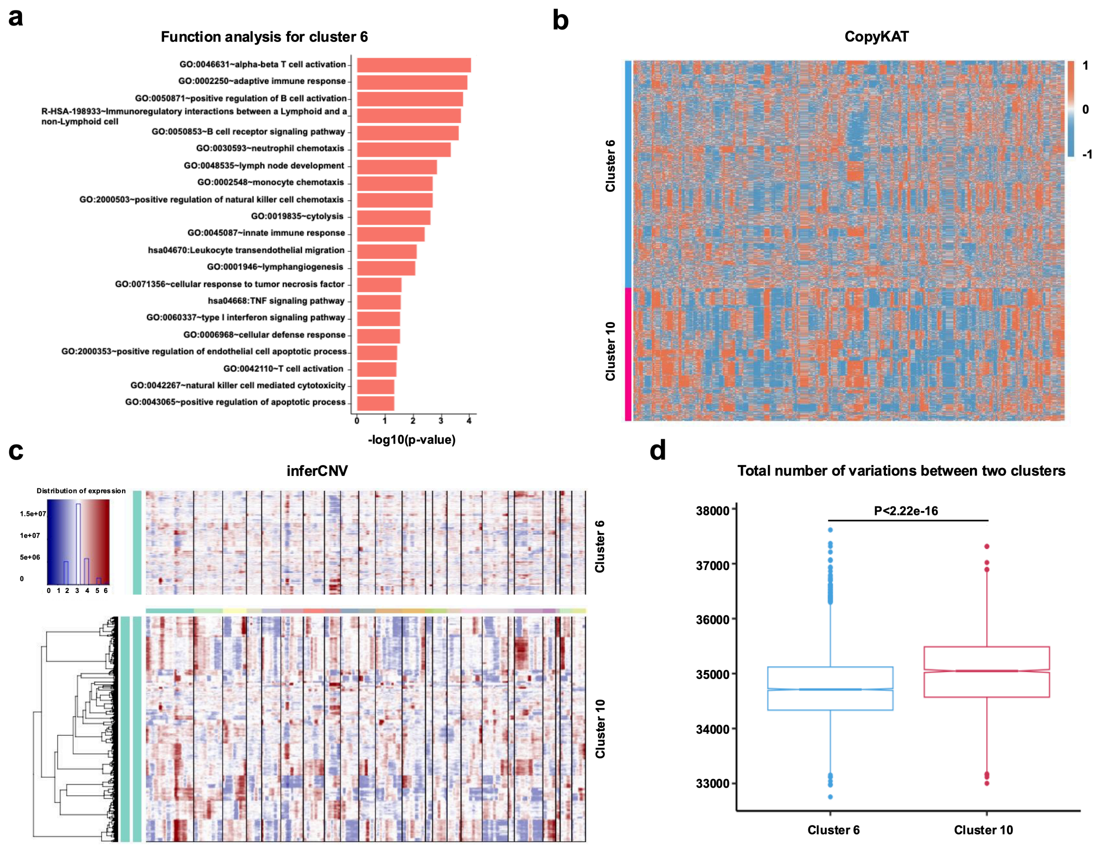

Supplementary Figure 17. Functional and copy number variation analyses of cluster 10 and cluster 6. a Functional annotation of over-expressed genes in cluster 6 identified by stClinic in the TNBC dataset. Unadjusted one-sided Fisher's exact test. **b** Copy number variations (CNVs) in clusters 6 and 10, estimated using CopyKAT [3]. **c** CNVs in clusters 6 ($N = 5{,}124$) and 10 ($N = 2{,}717$), estimated using inferCNV [4]. **d** Boxplot showing the total number of CNVs per spot for clusters 10 and cluster 6, as inferred by inferCNV. The $p-value$ was computed using unadjusted one-sided Wilcox rank-sum test. 2.22e-16 represents a very small numbers, effectively close to zero. The center line, box limits and whiskers for boxplot separately indicate the median, upper and lower quartiles and 1.5 × interquartile range. Source data are provided as a Source Data file.

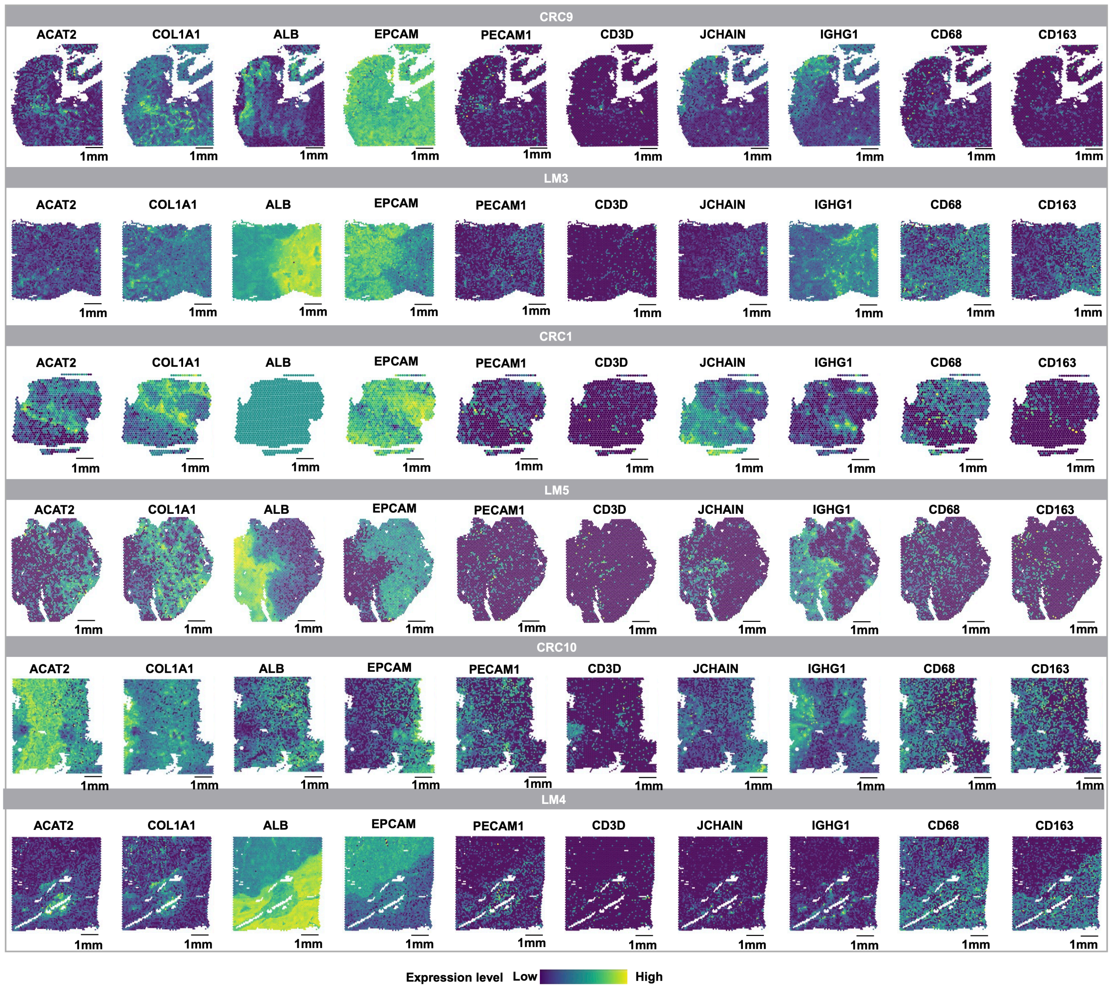

Supplementary Figure 18. Spatial expression of marker genes for stromal (*ACAT2*, and *COL1A1*), hepatocytes (*ALB*), epithelial (*EPCAM*), endothelial (*PECAM1*), T (*CD3D*), B (*JCHAIN*, and *IGHG1*), and myeloid cell (*CD68*, and *CD163*), for slices CRC9, LM3, CRC1, LM5, CRC10, and LM4 data denoised by stClinic. Source data are provided as a Source Data file.

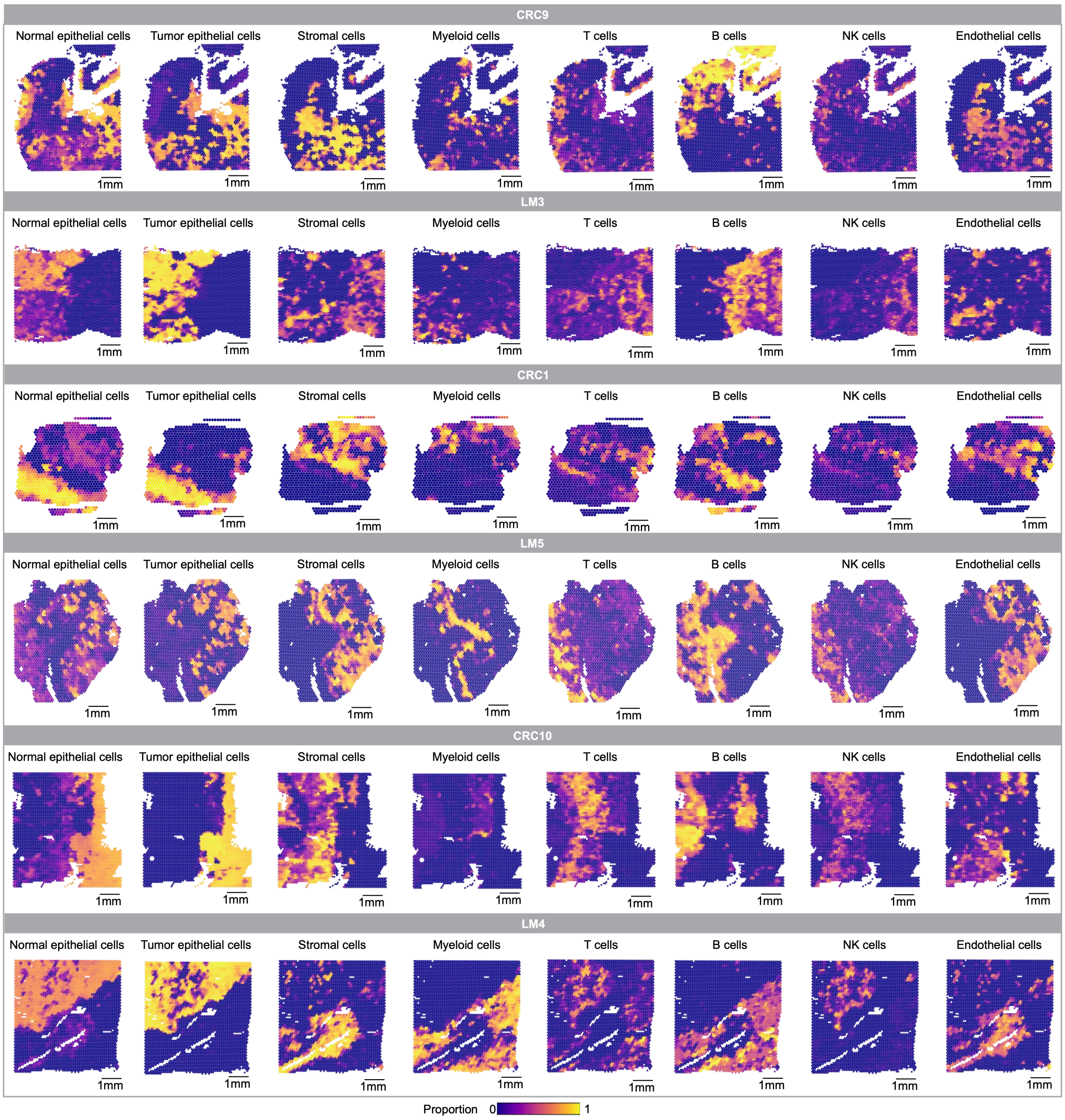

Supplementary Figure 19. Spatial distribution of eight cell types on slices CRC9, LM3, CRC1, LM5, CRC10, and LM4, respectively. Source data are provided as a Source Data file.

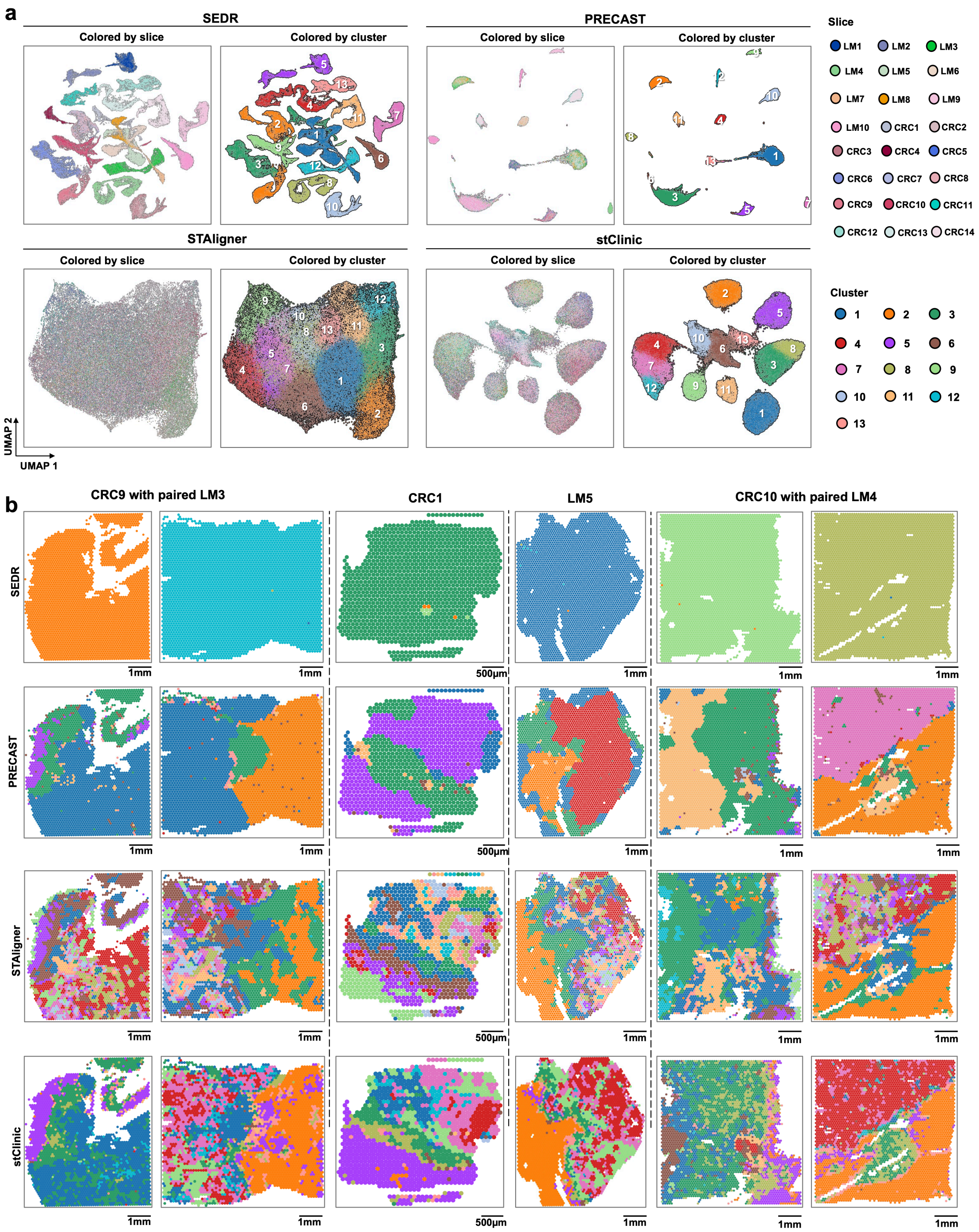

Supplementary Figure 20. Method comparison on 24 slices of the CRCLM dataset. **a** UMAP visualization of the latent features by SEDR, PRECAST, STAligner, and stClinic on 24 slices, where the colors in the left and right panels indicate slices and clusters, respectively. **b** Spatial domains identified by SEDR, PRECAST, STAligner, and stClinic on slices CRC9, LM3, CRC1, LM5, CRC10, and LM4, respectively. Source data are provided as a Source Data file.

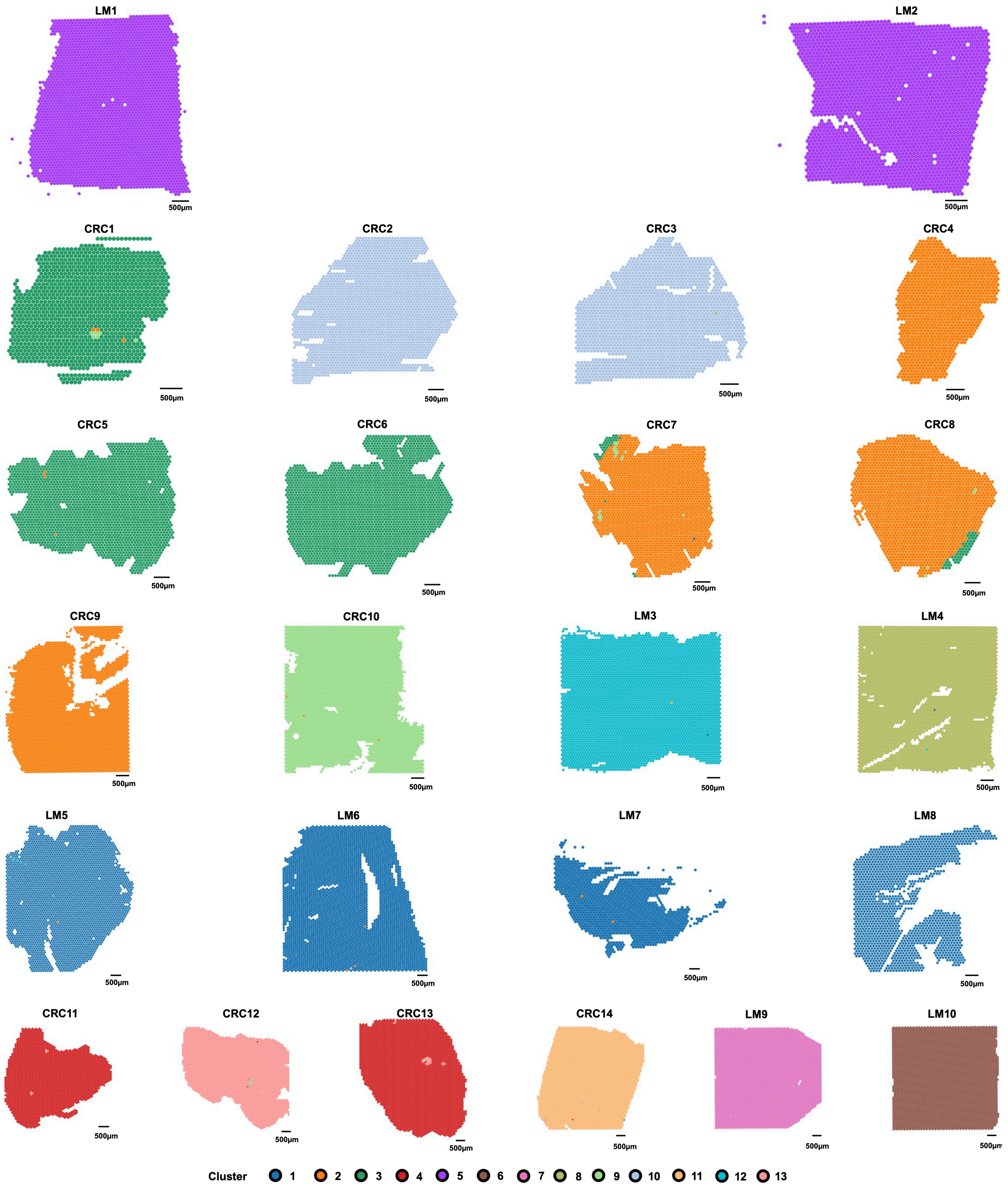

Supplementary Figure 21. Spatial domains identified by SEDR on 24 slices of the CRCLM dataset. Source data are provided as a Source Data file.

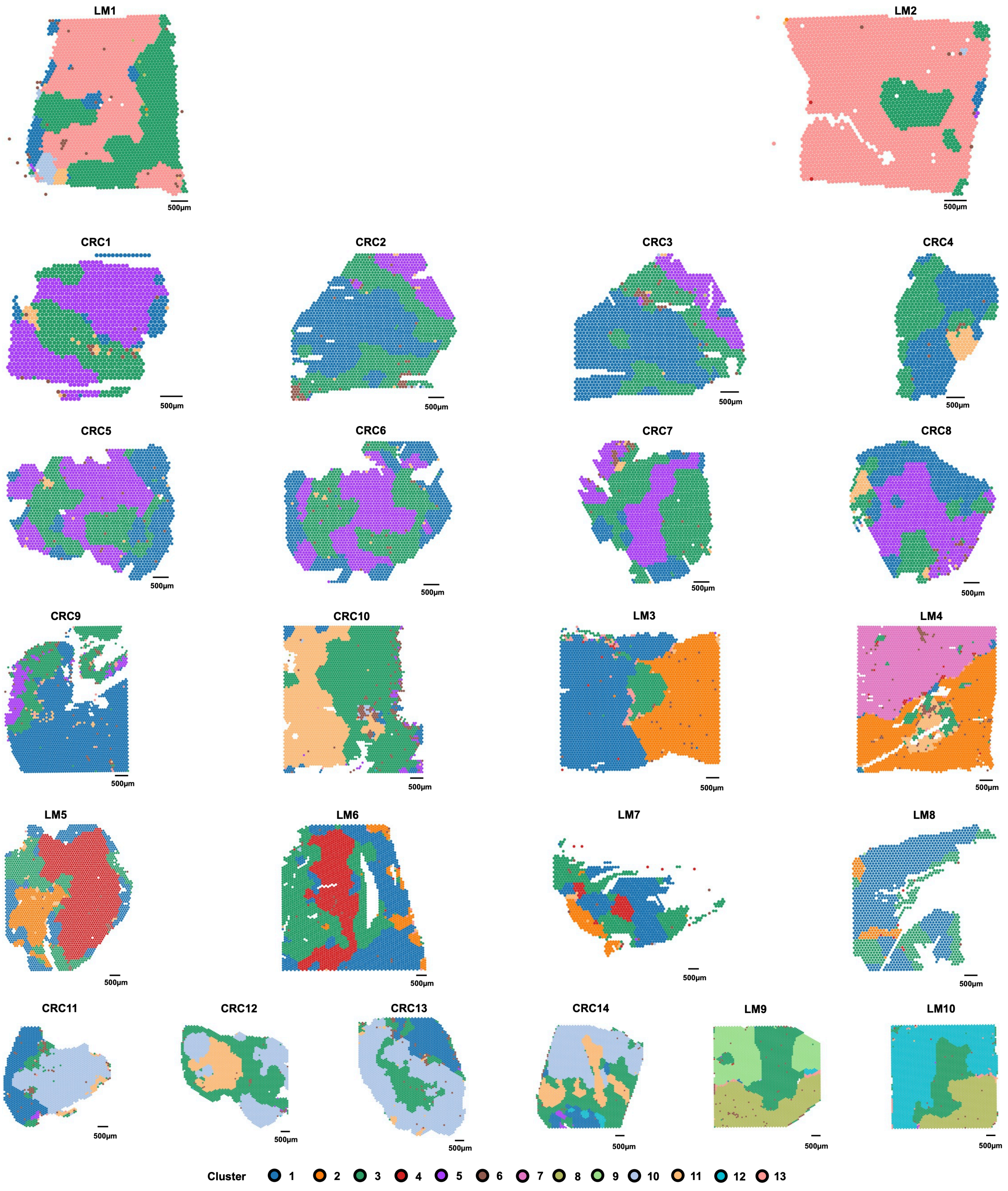

Supplementary Figure 22. Spatial domains identified by PRECAST on 24 slices of CRCLM dataset. Source data are provided as a Source Data file.

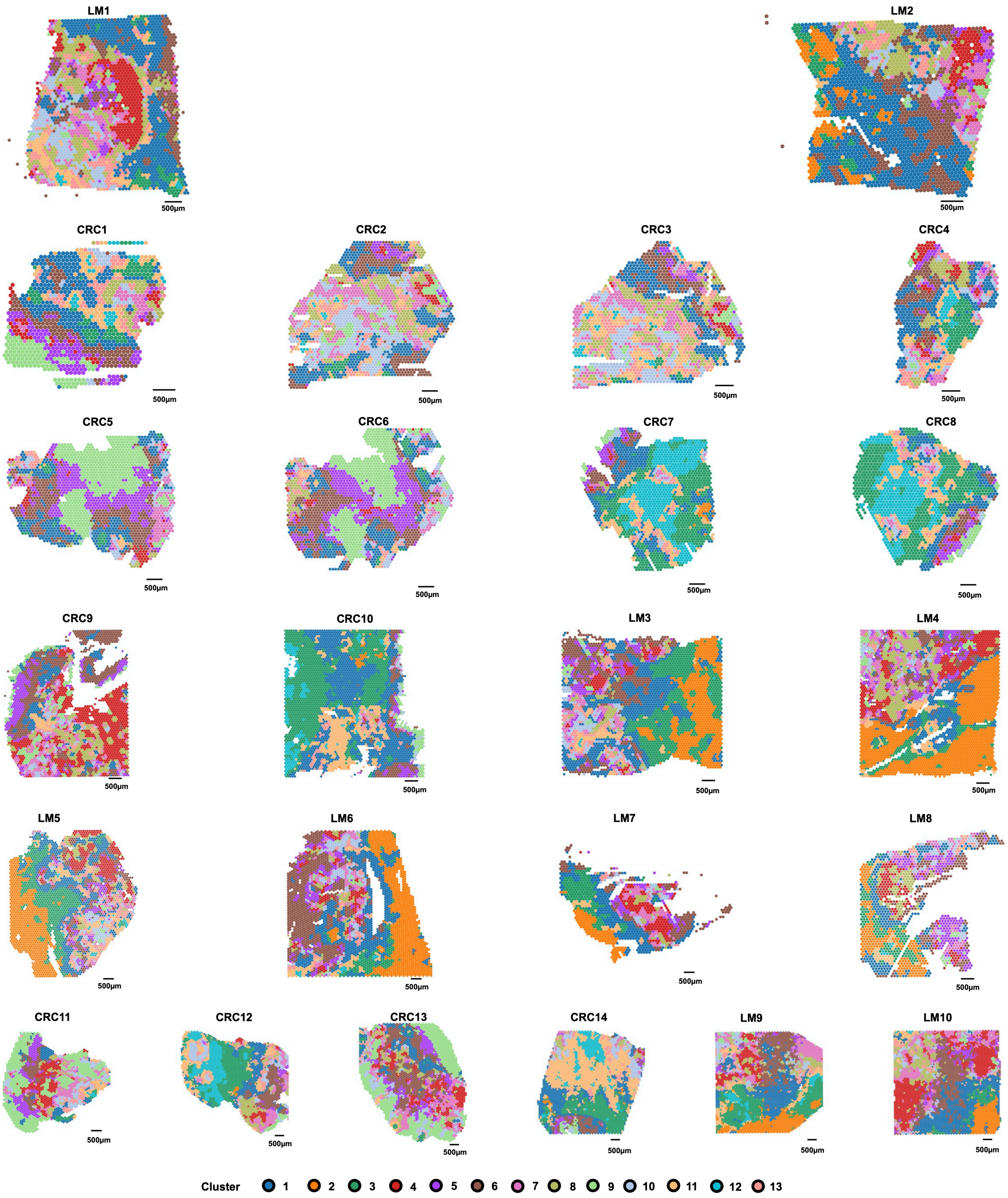

Supplementary Figure 23. Spatial domains identified by STAligner on 24 slices of the CRCLM dataset. Source data are provided as a Source Data file.

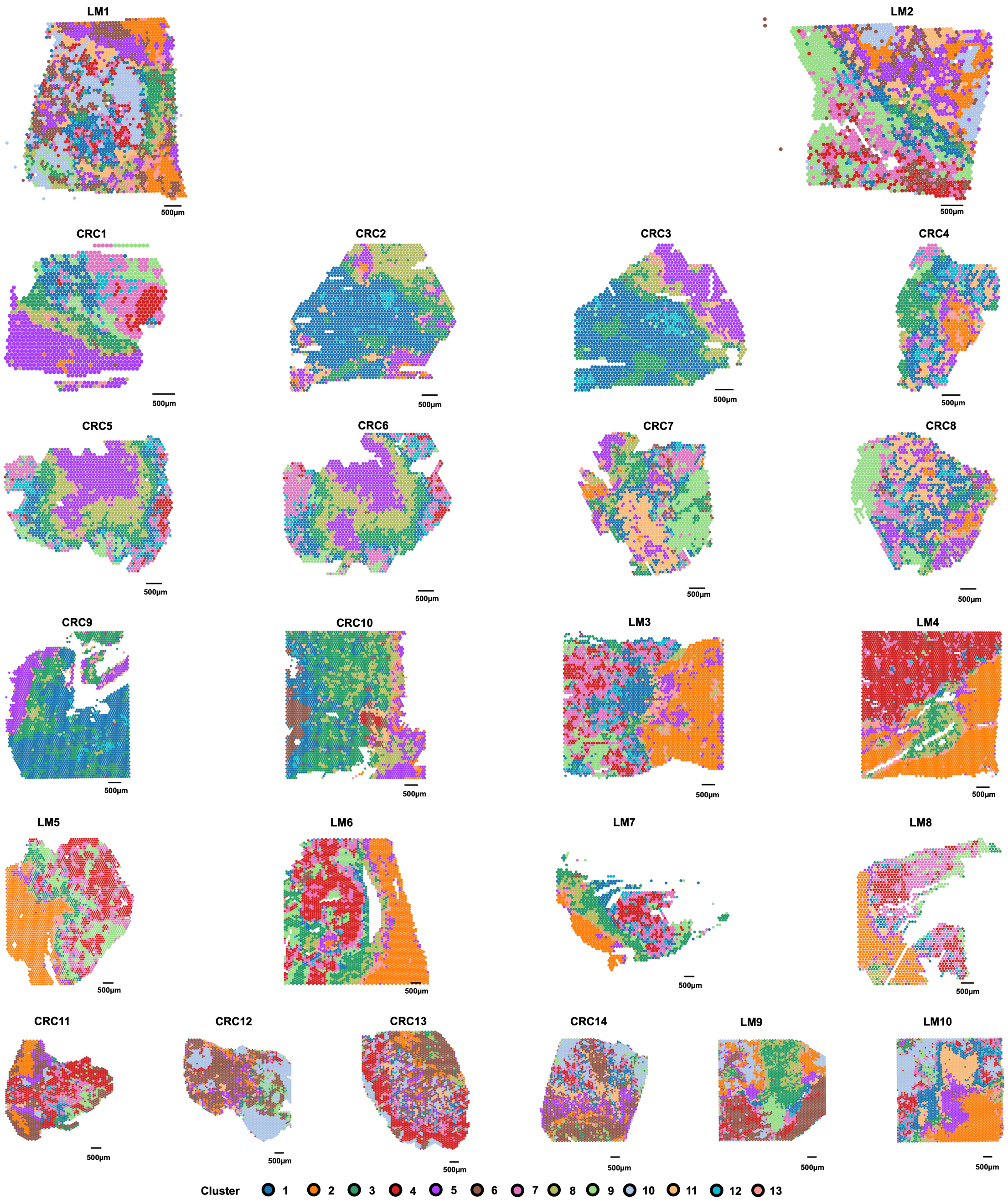

Supplementary Figure 24. Spatial domains identified by stClinic on 24 slices of the CRCLM dataset. Source data are provided as a Source Data file.

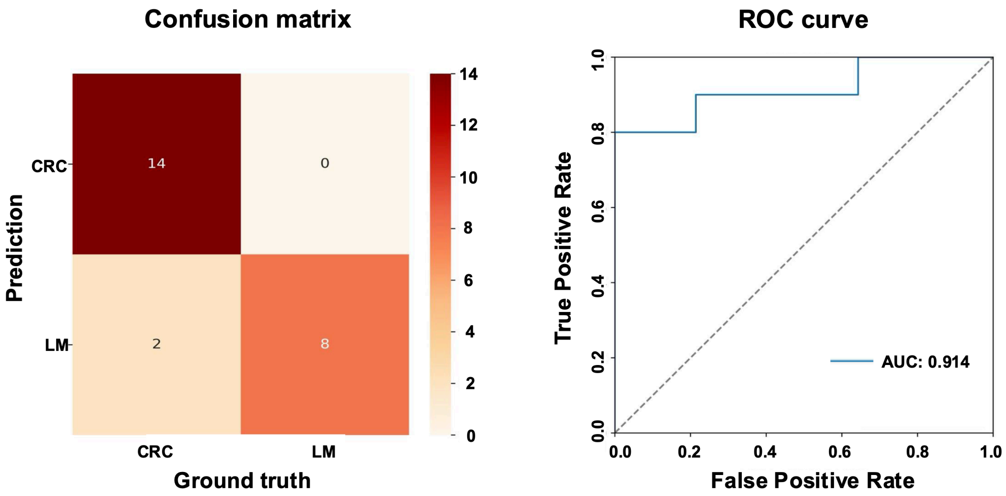

Supplementary Figure 25. The classification confusion matrix (left) along with the ROC curve (right) obtained by leave-one-out cross-validation (LOOCV) for stClinic, where 1 represents LM and 0 represents CRC in the CRCLM dataset. Source data are provided as a Source Data file.

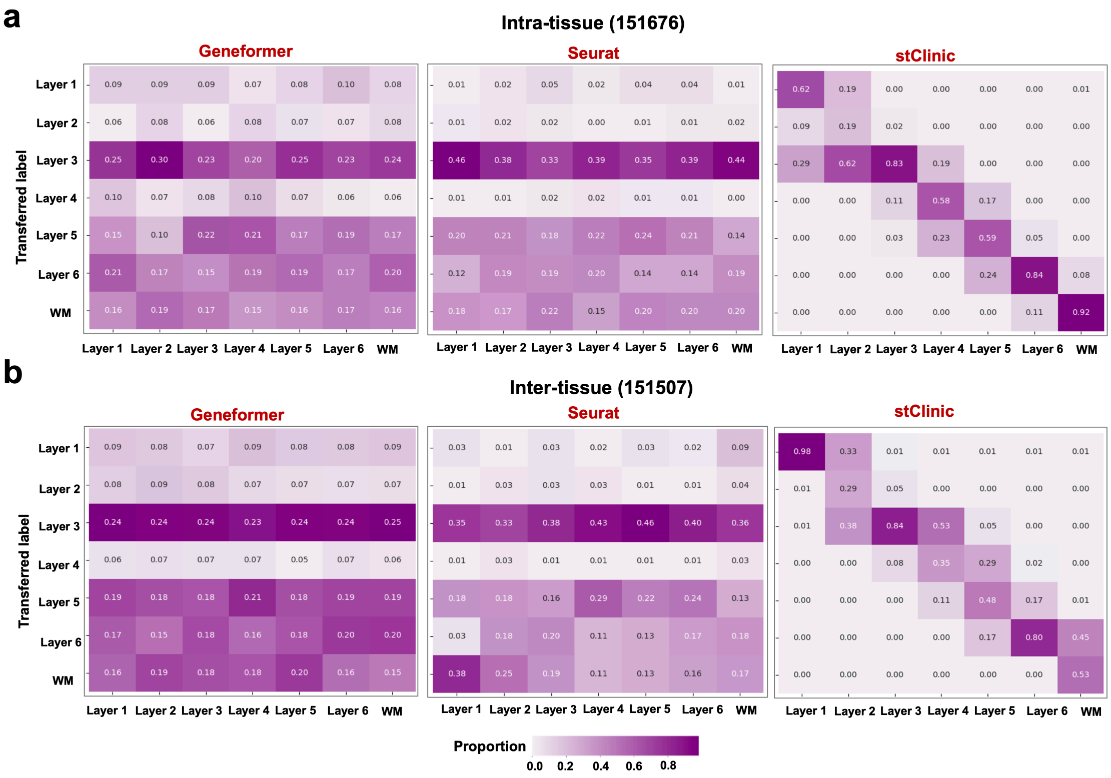

Supplementary Figure 26. Evaluation of zero-shot learning on the human DLPFC dataset. a Heatmap showing the proportion of interaction between each predicted cluster (by Geneformer, Seurat, and stClinic) and annotated cluster on slice 151676, compared to each annotated cluster. **b** Heatmap displaying the proportion of interaction between each predicted cluster (by Geneformer, Seurat, and stClinic) and annotated cluster on slice 151507, compared to each annotated cluster. Source data are provided as a Source Data file.

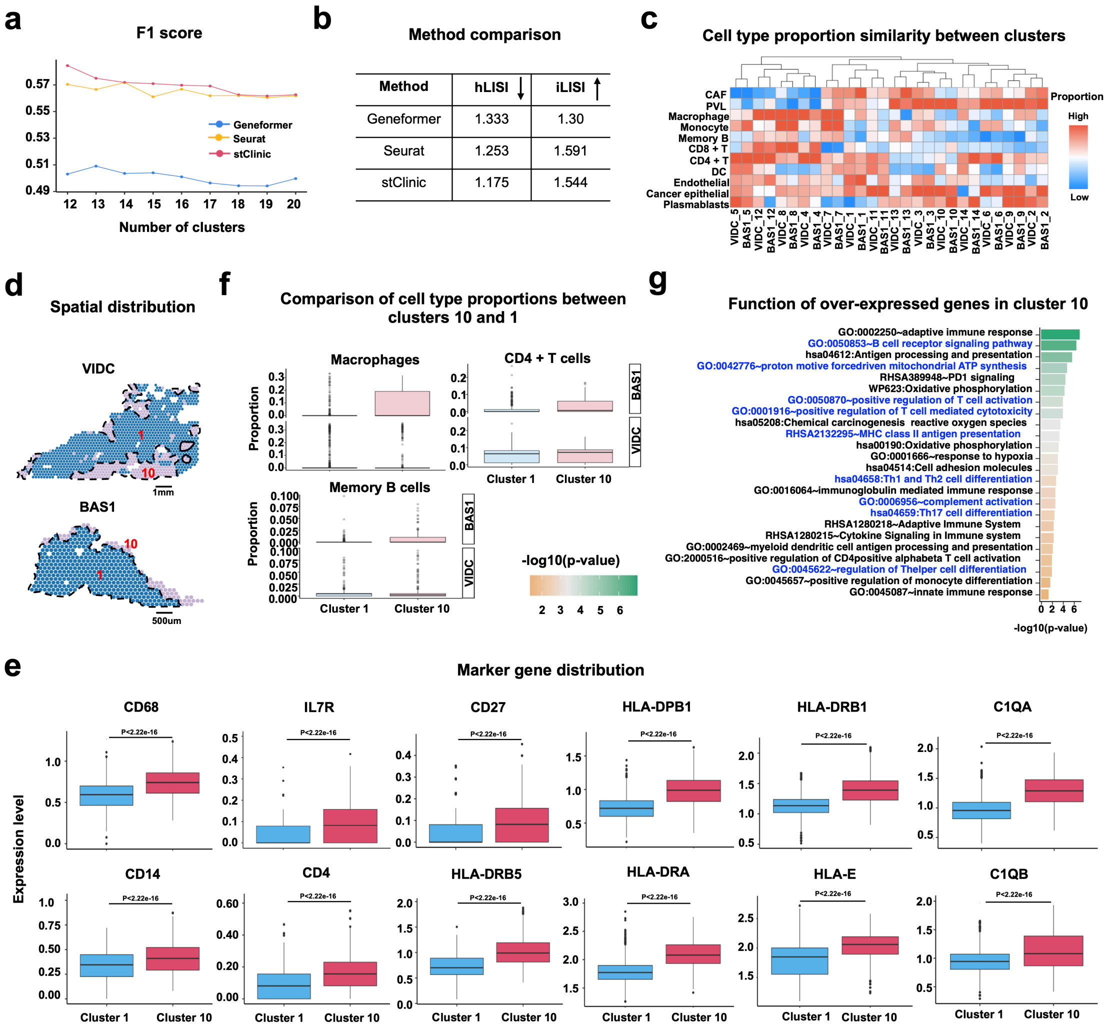

Supplementary Figure 27. Comparison of methods for the integration analysis of VIDC and BAS1 slices. a Evaluation of simultaneous cluster separation and slice mixing (F1 score) across varying numbers of clusters (12 to 20) for Geneformer, Seurat, and stClinic. **b** Slice mixing accuracy comparison of three methods based on histological annotation types (hLISI) and slice numbers (iLISI). **c** Hierarchical clustering of cell type proportion ranks within each cluster across two slices. **d** Spatial distribution of clusters 10 and 1. **e** Boxplot of expression levels of markers of macrophages (*CD68*, and *CD14*), CD4 + T cells (*CD4*, and *IL7R*), memory B cells (*CD27*), HLA class II molecules (*HLA-DRB5*, *HLA-DPB1*, *HLA-DRA*, *HLA-DRB1*, and *HLA-E*), and complement activation (*C1QA*, and *C1QB*) in cluster 10 (N =456) versus cluster 1 (N =1,375). Unadjusted one-sided Wilcox rank-sum test was used to compute $p - value$. 2.22e-16 represents a very small numbers, effectively close to zero. **f**

Boxplot comparing proportions of three cell types (macrophages, CD4 + T cells, and memory B cells) in cluster 10 (N =456) versus cluster 1 (N =1,375) across the two slices. **g** Functional annotation of over-expressed genes in cluster 10 relative to cluster 1. Unadjusted one-sided Fisher's exact test. For each boxplot of **e** and **f**, the center line, box limits and whiskers separately indicate the median, upper and lower quartiles and 1.5 × interquartile range. Source data are provided as a Source Data file.

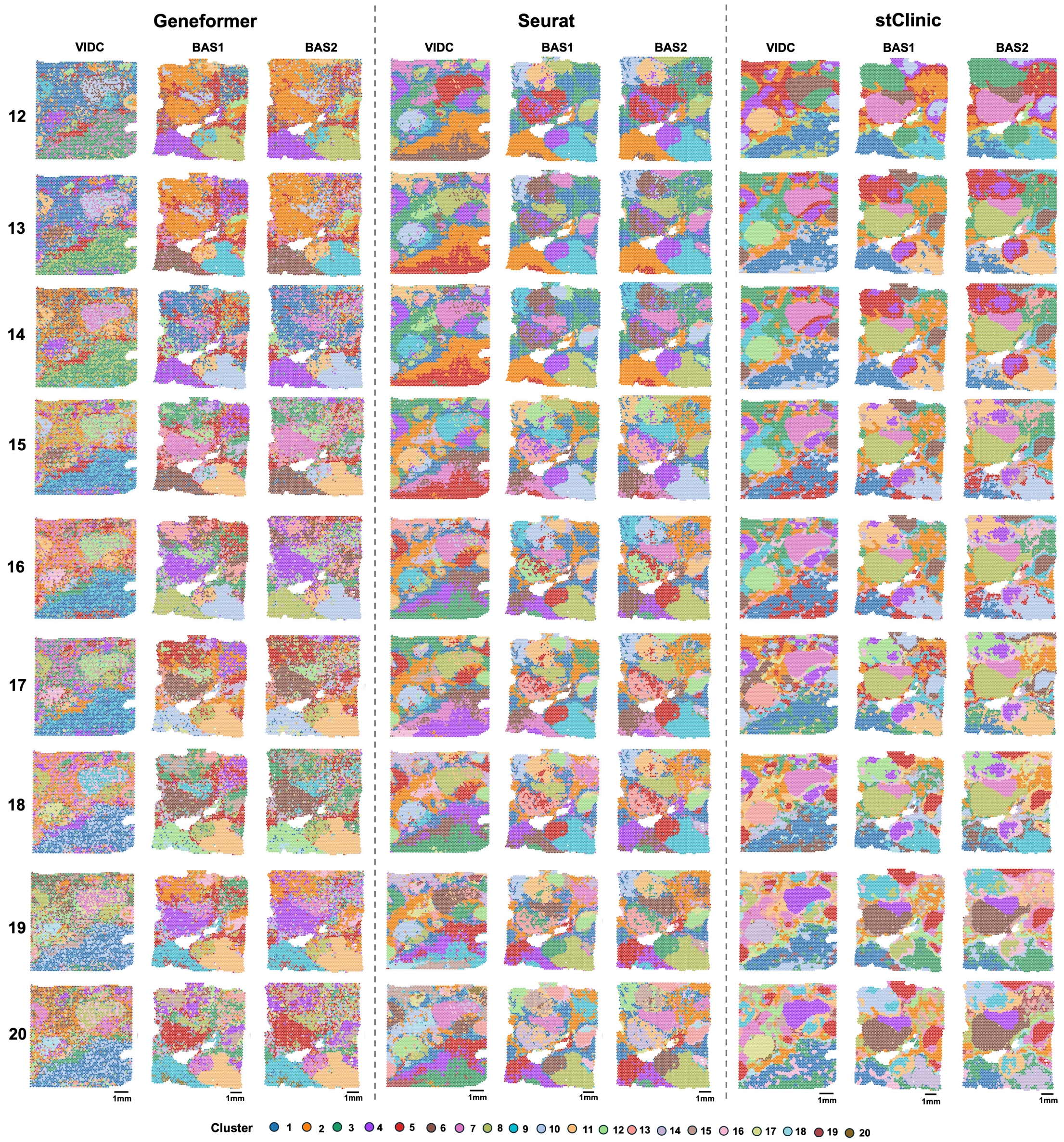

Supplementary Figure 28. Comparison of methods for the integration analysis of VIDC and BAS1 and label transfer to BAS2 across varying numbers of clusters (12 to 20) for Geneformer, Seurat, and stClinic. Source data are provided as a Source Data file.

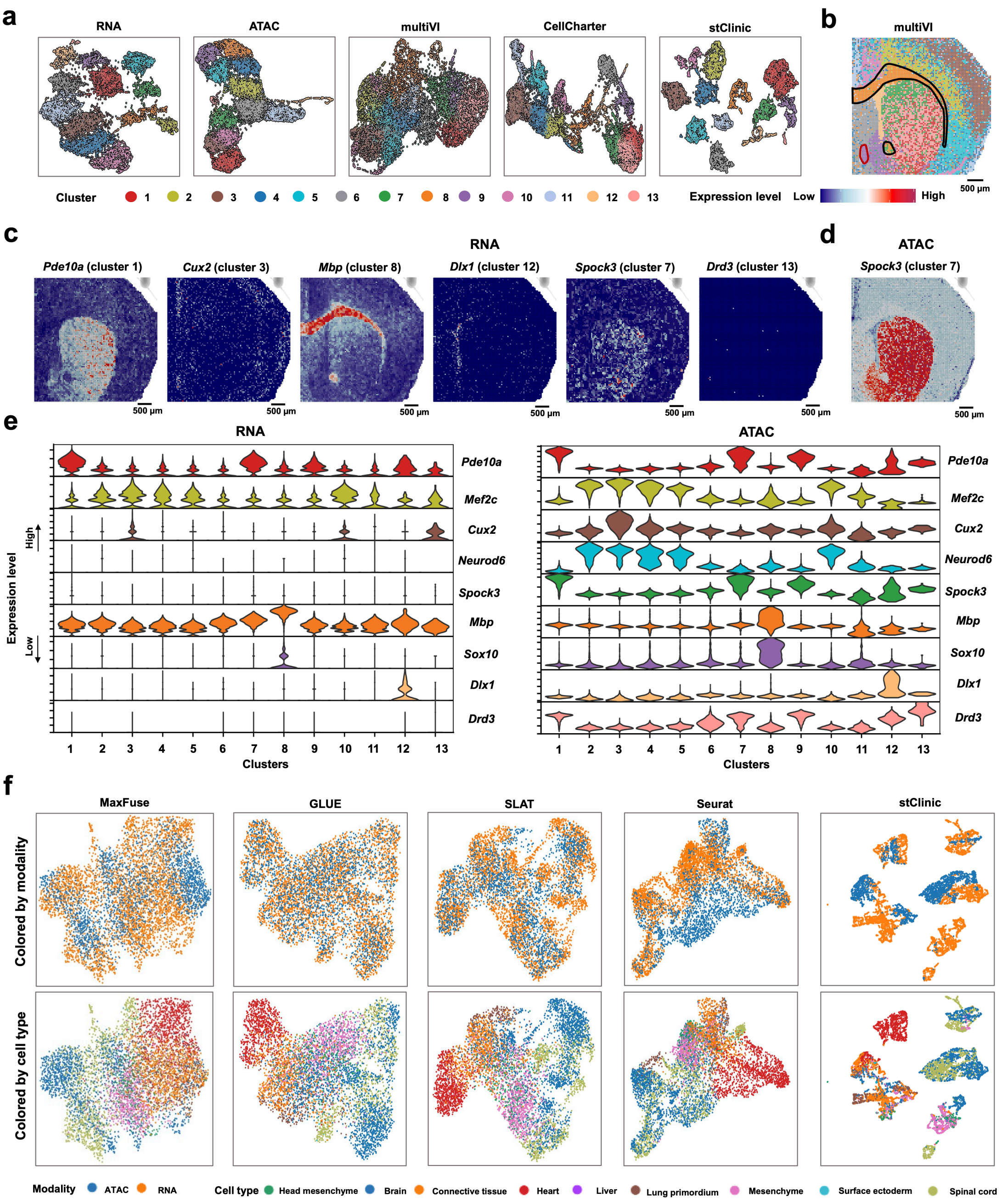

Supplementary Figure 29. Method comparison on spatial multi-omics dataset from the same slice and different slices. a UMAP visualization of the features by methods for single modality (RNA and ATAC), multi-modalities (multiVI, CellCharter, and stClinic), on mouse brain coronal section, respectively. Each color indicates one cluster. **b** Spatial domain identified by multiVI on mouse brain coronal section. **c** Spatial expression of marker genes for cluster 1 (*Pde10a*), cluster 3 (*Cux2*), cluster 7 (*Spock3*), cluster 8 (*Mbp*), cluster 12 (*Dlx1*), cluster 13 (*Drd3*), on mouse brain coronal section data denoised by stClinic. **d** Spatial ATAC levels of marker genes for cluster 7 (*Spock3*)

on mouse brain coronal section data denoised by stClinic. **e** Violin plot displaying the gene expression (left panel) and ATAC levels (right panel) of marker genes (*Pde10a*, *Mef2c*, *Cux2*, *Neurod6*, *Spock3*, *Mbp*, *Sox10*, *Dlx1*, and *Drd3*) in each cluster by stClinic, respectively. **f** UMAP visualization of the latent features by MaxFuse, GLUE, SLAT, Seurat, and stClinic on mouse embryo sample. In the top and bottom panels, the colors represent the modality and cluster, respectively. Source data are provided as a Source Data file.

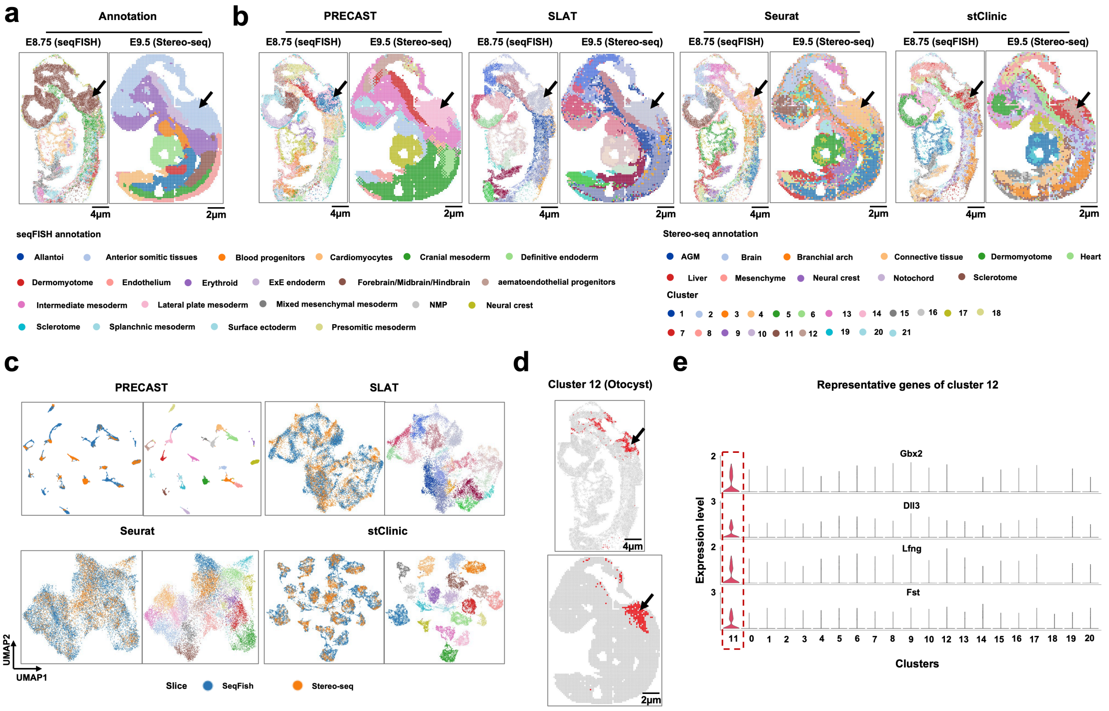

Supplementary Figure 30. Method comparison for integrative analysis of E8.75 seqFISH and E9.5 Stereo-seq mouse embryo slices. a Annotations of seqFISH and Stereo-seq from previous studies [5,6]. **b** Spatial domains identified by PRECAST, SLAT, Seurat, and stClinic for both slices. **c** UMAP visualization of features by PRECAST, SLAT, Seurat, and stClinic, respectively. For each method, the left panel shows color-coded slices, while right panel displays color-coded clusters. **d** Spatial distribution of cluster 12 (Otocyst) in both slices. **e** Violin plots showing gene expression of *Gbx2*, *Dll3*, *Lfng*, and *Fst* across different clusters. Source data are provided as a Source Data file.

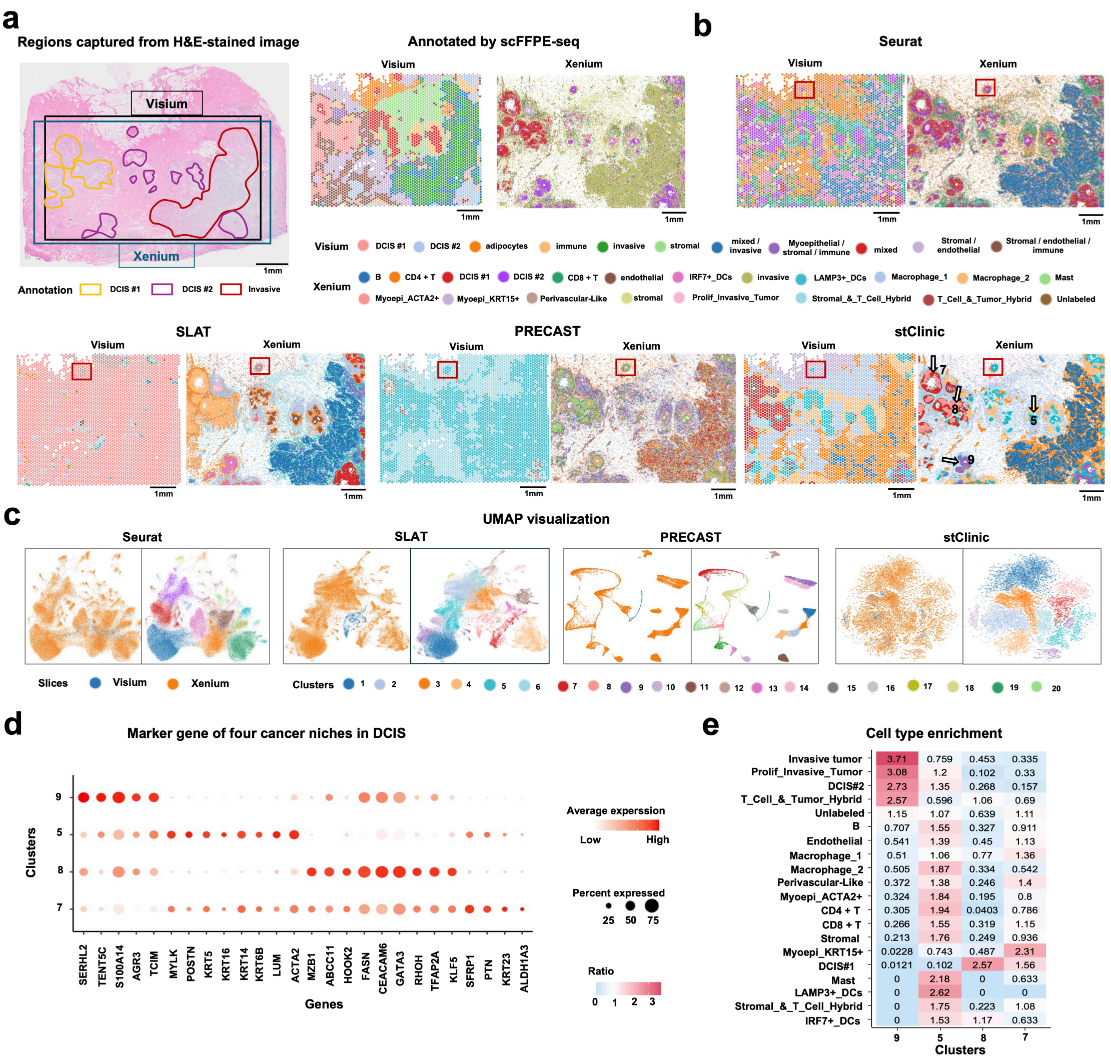

Supplementary Figure 31. Method comparison for integrative analysis of Visium and Xenium slices from the same breast cancer tissue. a Captured regions of the Visium and Xenium slices on the H&E image. **b** Spatial domains identified by PRECAST, SLAT, Seurat, and stClinic for both slices. **c** UMAP visualization of features by PRECAST, SLAT, Seurat, and stClinic, respectively. For each method, the left panel shows color-coded slices, while right panel displays color-coded clusters. **d** Dot plot showing the expression levels of marker genes across different niches in the DCIS region. **e** Heatmap displaying the relative enrichment of different cell-types across four niches. Source data are provided as a Source Data file.

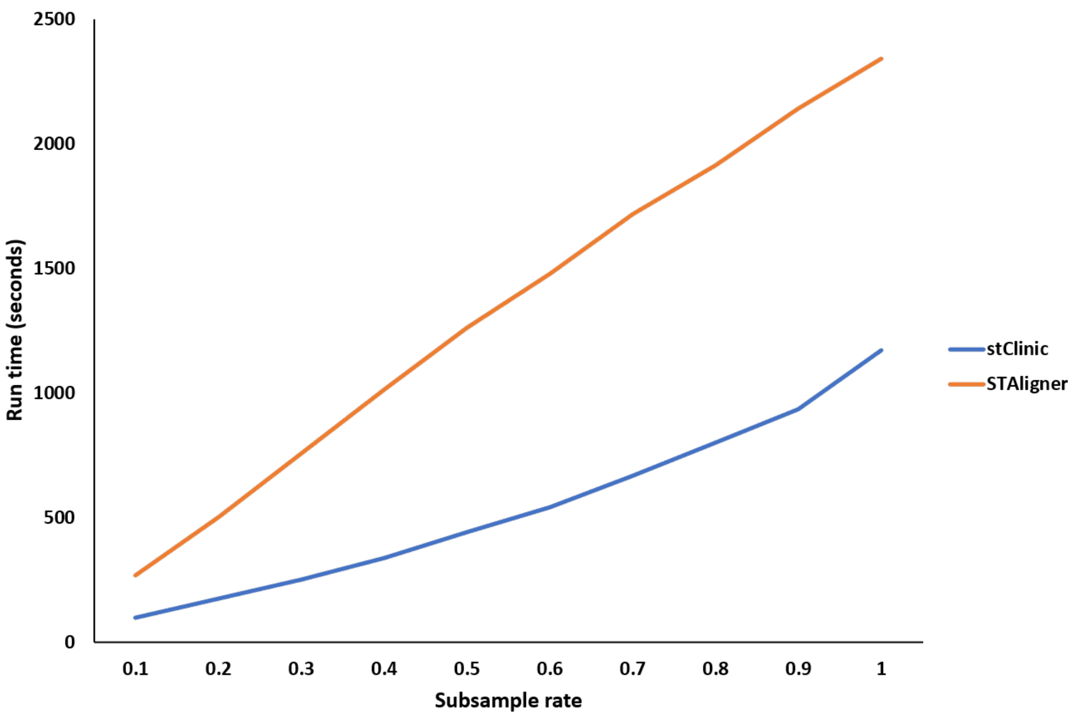

Supplementary Figure 32. Comparison of running time for the training of stClinic model in different number of spots by subsampling different proportions of spots from the primary CRC and LM dataset (68K spots). The experiments were tested on a GPU server with NVIDIA A800 GPU addressing 80GB. Source data are provided as a Source Data file.

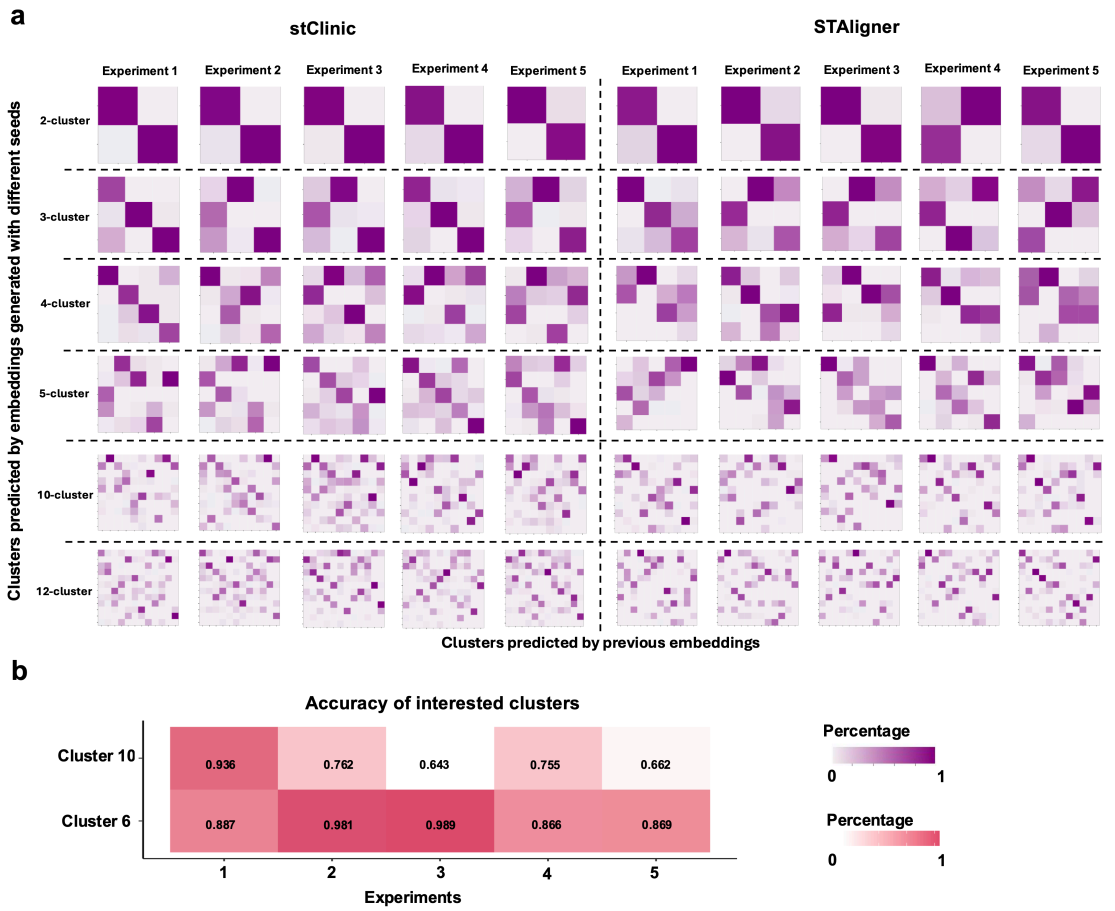

Supplementary Figure 33. Evaluation of prediction robustness on the TNBC dataset. a Heatmap showing the percentage of interactions between clusters predicted by latent features generated with different seeds (using stClinic and STAligner) and those predicted by latent features (as shown in Fig.4b and Supplementary Fig.8a), across different numbers of clusters. **b** Heatmap displaying the percentage of spots predicted as positive in cluster 10 and negative in cluster 6 across five experiments with different seeds. Source data are provided as a Source Data file.

Sample	Tissue	Technology	# Slice	# Cell / spot	Reference	Download link
DLPFC	Human dorsolateral prefrontal cortex	10X Visium	12	47,329	[7]	https://github.com/LieberInstitute/spatialLIBD
3D Hippo	Mouse hippocampal structure	Slide-seq	7	10908	[8]	https://drive.google.com/drive/folders/10lhz5VY7YfvHrtV40MwaqLmWz56U9eBP?usp=sharing
VIDC	Human Luminal B breast cancer	10X Visium	1	4727	[9]	https://www.10xgenomics.com/datasets/invasive-ductal-carcinoma-stained-with-fluorescent-cd-3-antibody-1-standard-1-2-0
BAS1		10X Visium	1	3798		https://www.10xgenomics.com/datasets/human-breast-cancer-block-a-section-1-1-standard-1-1-0
BAS2		10X Visium	1	3987		https://www.10xgenomics.com/datasets/human-breast-cancer-block-a-section-1-1-standard-1-1-0
CID4535	Human Luminal B breast cancer	scRNA-seq	1	3961	[10]	https://www.ncbi.nlm.nih.gov/geo/query/acc.cgi?acc=GSE176078
Visium	Her2+ human breast cancer	10X Visium	1	3841	[11]	https://www.ncbi.nlm.nih.gov/geo/query/acc.cgi?acc=GSE243280
Xenium		10X Xenium	1	100642		
seqFISH	E8.75 mouse embryo	seqFISH	1	>10000	[5]	https://marionilab.cruk.cam.ac.uk/SpatialMouseAtlas/
Stereo-seq	E9.5 mouse embryo	Stereo-seq	1	5000	[6]	https://db.cngb.org/stomics/mosta/download/

TNBC	Human triple-negative breast cancer	10X Visium	43	56,567	[12]	https://www.ncbi.nlm.nih.gov/geo/query/acc.cgi?acc=GSE210616
		Clinical data	43	NA		https://doi.org/10.1158/0008-5472.CAN-22-2682
TNBC	Human triple-negative breast cancer	scRNA-seq	10	42,512	[10]	https://www.ncbi.nlm.nih.gov/geo/query/acc.cgi?acc=GSE176078
CRCLM	Human colorectal cancers and liver metastases	10X Visium	24	68,261	[13-17]	https://drive.google.com/file/d/1QsQIT0-iwcWBFzUBcLUPKYuSnSBxfaME/view?usp=drive_link
CRC	Human colorectal cancers	scRNA-seq	1	6,275	[18]	https://www.ncbi.nlm.nih.gov/geo/query/acc.cgi?acc=GSE132465
Brain	P2 mouse brain coronal section	Spatial ATAC-RNA-seq	1	9,215	[19]	https://www.ncbi.nlm.nih.gov/geo/query/acc.cgi?acc=GSE205055
Embryo	E10.5 mouse embryo	Stereo-seq	1	4,132	[20]	https://db.cngb.org/stomics/mosta/download/
	E11 mouse embryo	Spatial ATAC-seq	1	2,099	[6]	https://www.ncbi.nlm.nih.gov/geo/query/acc.cgi?acc=GSE171943

Supplementary Table 1. Summary of the used dataset.

Application	Method	Clustering method	Pipeline
Integrative analysis of the DLPFC dataset	SEDR	mclust	https://sedr.readthedocs.io/en/latest/Tutorial3_Batch_integration.html
	GraphST	mclust	https://deepst-tutorials.readthedocs.io/en/latest/Tutorial%205_Vertical%20Integration.html
	Stitch3D	Gaussian Mixture Model	https://stitch3d-tutorial.readthedocs.io/en/latest/tutorials/DLPFC/STitch3D_DLPFC.html
	PRECAST	Built-in spatial factor model	https://feiyoung.github.io/PRECAST/articles/PRECAST.DLPFC4.html
	STAligner	mclust	https://staligner.readthedocs.io/en/latest/Tutorial_DLPFC.html
	stClinic	mclust	https://github.com/cmzuo11/stClinic/
Integrative analysis of tumor samples	SpaGCN	Louvain	https://github.com/jianhuupenn/SpaGCN/blob/master/tutorial/tutorial.ipynb
	SLAT	Leiden	https://slat.readthedocs.io/en/latest/tutorials/cross_technology.html
	Seurat	Louvain	https://satijalab.org/seurat/articles/spatial_vignette
	SEDR	mclust	https://sedr.readthedocs.io/en/latest/Tutorial3_Batch_integration.html
	STAligner	Louvain	https://staligner.readthedocs.io/en/latest/Tutorial_embryo.html
	PRECAST	Built-in spatial factor model	https://feiyoung.github.io/PRECAST/articles/PRECAST.BreastCancer.html
	BANKSY	Leiden	https://prabhakarlab.github.io/Banksy/articles/multi-sample.html
	stClinic	Louvain	https://github.com/cmzuo11/stClinic/
Label Transfer	Geneformer	Louvain	https://geneformer.readthedocs.io/en/latest/getstarted.html and https://chanzuckerberg.github.io/cellxgene-census/notebooks/analysis_demo/comp_bio_geneformer_prediction.html
	Seurat	Louvain	https://satijalab.org/seurat/articles/integration_mapping

	stClinic	Louvain	https://github.com/cmzuo11/stClinic/

Supplementary Table 2. Summary of clustering methods and pipelines used for comparison across different experiments.

Layers	Genes
Layer 1	*FABP7*, *RELN*
Layer 2	*HPCAL1*
Layer 3	*CARTPT*, *FREM3*
Layer 4	*PVALB*, *RORB*
Layer 5	*TRABD2A*, *PCP4*
Layer 6	*KRT17*, *NTNG2*
WM	*MOBP*, *MBP*

Supplementary Table 3. Known layer-specific genes for the human DLPFC dataset from a previous study [7].

References

1 Lu, M. Y. *et al.* A visual-language foundation model for computational pathology. *Nature Medicine* **30**, 863-874, doi:10.1038/s41591-024-02856-4 (2024).

2 Long, Y. *et al.* Spatially informed clustering, integration, and deconvolution of spatial transcriptomics with GraphST. *Nat Commun* **14**, 1155 (2023).

3 Gao, R. *et al.* Delineating copy number and clonal substructure in human tumors from single-cell transcriptomes. *Nature biotechnology* **39**, 599-608 (2021).

4 Erickson, A. *et al.* Spatially resolved clonal copy number alterations in benign and malignant tissue. *Nature* **608**, 360-367, doi:10.1038/s41586-022-05023-2 (2022).

5 Lohoff, T. *et al.* Integration of spatial and single-cell transcriptomic data elucidates mouse organogenesis. *Nature biotechnology* **40**, 74-85 (2022).

6 Chen, A. *et al.* Spatiotemporal transcriptomic atlas of mouse organogenesis using DNA nanoball-patterned arrays. *Cell* **185**, 1777-1792. e1721 (2022).

7 Maynard, K. R. *et al.* Transcriptome-scale spatial gene expression in the human dorsolateral prefrontal cortex. *Nature neuroscience* **24**, 425-436 (2021).

8 Rodriques, S. G. *et al.* Slide-seq: A scalable technology for measuring genome-wide expression at high spatial resolution. *Science* **363**, 1463-1467 (2019).

9 10xgenomics. *Explore and download datasets created by 10x Genomics.*, <https://www.10xgenomics.com/datasets> (2024).

10 Wu, S. Z. *et al.* A single-cell and spatially resolved atlas of human breast cancers. *Nature Genetics* **53**, 1334-1347, doi:10.1038/s41588-021-00911-1 (2021).

11 Janesick, A. *et al.* High resolution mapping of the tumor microenvironment using integrated single-cell, spatial and in situ analysis. *Nat Commun* **14**, 8353 (2023).

12 Bassiouni, R. *et al.* Spatial transcriptomic analysis of a diverse patient cohort reveals a conserved architecture in triple-negative breast cancer. *Cancer research* **83**, 34-48 (2023).

13 Valdeolivas, A. *et al.* Charting the heterogeneity of colorectal cancer consensus molecular subtypes using spatial transcriptomics. *bioRxiv*, 2023.2001. 2023.525135 (2023).

14 Villemin, J.-P. *et al.* Inferring ligand-receptor cellular networks from bulk and spatial transcriptomic datasets with BulkSignalR. *Nucleic Acids Res* **51**, 4726-4744 (2023).

15 Wang, F. *et al.* Single-cell and spatial transcriptome analysis reveals the cellular heterogeneity of liver metastatic colorectal cancer. *Science advances* **9**, eadf5464 (2023).

16 Garbarino, O. *et al.* Spatial resolution of cellular senescence dynamics in human colorectal liver metastasis. *Aging Cell* **22**, e13853 (2023).

17 Wu, Y. *et al.* Spatiotemporal immune landscape of colorectal cancer liver metastasis at single-cell level. *Cancer discovery* **12**, 134-153 (2022).

18 Lee, H.-O. *et al.* Lineage-dependent gene expression programs influence the immune landscape of colorectal cancer. *Nature Genetics* **52**, 594-603, doi:10.1038/s41588-020-0636-z (2020).

19 Zhang, D. *et al.* Spatial epigenome–transcriptome co-profiling of mammalian tissues. *Nature* **616**, 113-122 (2023).

20 Deng, Y. *et al.* Spatial profiling of chromatin accessibility in mouse and human tissues. *Nature* **609**, 375-383 (2022).
